# Therapeutic Efficacy of the Inositol D-Pinitol as a Multi-Faceted Disease Modifier in the 5×FAD Humanized Mouse Model of Alzheimer’s Amyloidosis

**DOI:** 10.3390/nu16234186

**Published:** 2024-12-04

**Authors:** Dina Medina-Vera, Antonio J. López-Gambero, Julia Verheul-Campos, Juan A. Navarro, Laura Morelli, Pablo Galeano, Juan Suárez, Carlos Sanjuan, Beatriz Pacheco-Sánchez, Patricia Rivera, Francisco J. Pavon-Morón, Cristina Rosell-Valle, Fernando Rodríguez de Fonseca

**Affiliations:** 1 Grupo de Neuropsicofarmacología, Instituto de Investigación Biomédica de Málaga y Plataforma en Nanomedicina-IBIMA Plataforma BIONAND, Unidades Clínicas de Neurología y Salud Mental, 29010 Málaga, Spain; antonio.lopez@ibima.eu (A.J.L.-G.); verheuljulia@gmail.com (J.V.-C.); juan_naga@hotmail.es (J.A.N.); juan.suarez@ibima.eu (J.S.); beatriz.pacheco@ibima.eu (B.P.-S.); patricia.rivera@ibima.eu (P.R.); javier.pavon@ibima.eu (F.J.P.-M.); 2 Facultad de Ciencias, Universidad de Málaga, 29010 Málaga, Spain; 3 Unidad de Gestión Clínica del Corazón—CIBERCV (Enfermedades Cardiovasculares), Hospital Universitario Virgen de la Victoria, 29010 Málaga, Spain; 4 INSERM, Neurocentre Magendie, University of Bordeaux, 33000 Bordeaux, France; 5 Facultad de Medicina, Universidad de Málaga, 29010 Málaga, Spain; 6 Laboratory of Brain Aging and Neurodegeneration, Fundación Instituto Leloir (IIBBA-CONICET), Av. Patricias Argentinas 435, Ciudad Autónoma de Buenos Aires C1405BWE, Argentina; lmorelli@leloir.org.ar (L.M.); pgaleano@leloir.org.ar (P.G.); 7 Departamento de Anatomía Humana, Medicina Legal e Historia de la Ciencia, Facultad de Medicina, Universidad de Málaga, 29071 Málaga, Spain; 8 Andalusian Network for Clinical and Translational Research in Neurology [NEURO-RECA], 29001 Málaga, Spain; 9 Euronutra S.L. Calle Johannes Kepler, 3, 29590 Málaga, Spain; euronutra@euronutra.eu

**Keywords:** Alzheimer’s disease, brain insulin resistance, hippocampus, Aβ plaques, CDK5, microbiota dysbiosis, tau phosphorylation

## Abstract

Background/Objectives: Alzheimer’s disease (AD), a leading cause of dementia, lacks effective long-term treatments. Current therapies offer temporary relief or fail to halt its progression and are often inaccessible due to cost. AD involves multiple pathological processes, including amyloid beta (Aβ) deposition, insulin resistance, tau protein hyperphosphorylation, and systemic inflammation accelerated by gut microbiota dysbiosis originating from a leaky gut. Given this context, exploring alternative therapeutic interventions capable of addressing the multifaceted components of AD etiology is essential. Methods: This study suggests D-Pinitol (DPIN) as a potential treatment modifier for AD. DPIN, derived from carob pods, demonstrates insulin-sensitizing, tau hyperphosphorylation inhibition, and antioxidant properties. To test this hypothesis, we studied whether chronic oral administration of DPIN (200 mg/kg/day) could reverse the AD-like disease progression in the 5×FAD mice. Results: Results showed that treatment of 5×FAD mice with DPIN improved cognition, reduced hippocampal Aβ and hyperphosphorylated tau levels, increased insulin-degrading enzyme (IDE) expression, enhanced pro-cognitive hormone circulation (such as ghrelin and leptin), and normalized the PI3K/Akt insulin pathway. This enhancement may be mediated through the modulation of cyclin-dependent kinase 5 (CDK5). DPIN also protected the gut barrier and microbiota, reducing the pro-inflammatory impact of the leaky gut observed in 5×FAD mice. DPIN reduced bacterial lipopolysaccharide (LPS) and LPS-associated inflammation, as well as restored intestinal proteins such as Claudin-3. This effect was associated with a modulation of gut microbiota towards a more balanced bacterial composition. Conclusions: These findings underscore DPIN’s promise in mitigating cognitive decline in the early AD stages, positioning it as a potential disease modifier.

## 1. Introduction

Treating Alzheimer’s disease (AD), the leading cause of severe cognitive impairment and dementia, remains an unresolved clinical challenge. Current medications, including recently approved immunotherapies [1], provide temporary symptom relief but fail to halt disease progression and are inaccessible for many patients. AD is a multifactorial condition involving diverse pathological processes beyond amyloid beta (Aβ) and tau protein deposition [2], such as central insulin resistance [3], systemic inflammation, and gut microbiota alterations [4,5,6]. Addressing this complexity requires exploring therapies targeting multiple mechanisms simultaneously.

Insulin resistance, a hallmark of type 2 diabetes, has been strongly linked to AD [7,8], prompting some authors to label it as “type 3 diabetes” [9]. In the central nervous system (CNS), insulin resistance reduces insulin’s effect, disrupting glucose metabolism and leading to energy deficits in brain cells. These energy deficits compromise neuronal function, impair synaptic plasticity, alter neurotransmitter release, and increase vulnerability to oxidative stress [10].

Insulin resistance also influences key AD pathologies through the PI3K/Akt pathways [11,12]. Dysregulation of PI3K/Akt pathway due to insulin resistance increases the risk of AD [13,14,15,16]. Dysfunctional insulin signaling influences critical processes in AD beyond neurofibrillary tangles [17,18,19] and Aβ plaques [20], including neuronal survival and Aβ metabolism [21]. Additionally, the cyclin-dependent ksssinase 5 (CDK5) has emerged as a key regulator in AD pathology [22]. Post-mortem analysis of AD patients shows an accumulation of p25 and increased CDK5 activity, contributing to neurodegeneration [23,24]. Moreover, hyperinsulinemia may impact extracellular Aβ levels by affecting insulin-degrading enzyme (IDE), preferentially degrading insulin over Aβ [25].

The gut microbiota, a complex microbial ecosystem essential for immune regulation [26,27] and brain health [28], plays a significant role in AD pathogenesis [29,30]. Emerging evidence underscores its impact also on neuronal plasticity [31], and cognitive function [32,33]. Dysbiosis, or microbial imbalance, can lead to systemic inflammation [34], increased lipopolysaccharide (LPS) production [35], and a weakened blood–brain barrier (BBB) [36,37], all of which aggravate neuroinflammation and cognitive decline in AD [35]. These findings underscore the therapeutic potential of restoring microbial balance to mitigate AD progression [38,39]. Restoring microbial balance and improving intestinal barrier function may positively impact AD progression and CNS health.

In this context, exploring therapeutic interventions that target multiple aspects of AD pathology is crucial. This study aims to evaluate D-Pinitol (DPIN) as a potential therapeutic candidate. DPIN, a natural inositol derived from carob pods, has demonstrated insulin-sensitizing properties and can facilitate tau protein dephosphorylation by modulating CDK5 kinase activity [40,41]. Recognized for its therapeutic potential [42], DPIN acts as an insulin sensitizer, enhancing insulin signaling and mitigating peripheral insulin resistance [43,44]. Although direct research on DPIN’s effects on the gut microbiota is limited, its insulin-sensitizing properties and ability to counter insulin resistance [42,43,45] suggest a potential indirect influence on gut microbial composition. By targeting insulin resistance and related pathways [46], DPIN represents a promising candidate for addressing the multifaceted pathology of AD.

Building on prior research involving DPIN [41,44,47], this study evaluates the therapeutic potential of DPIN in the 5×FAD mouse model, which replicates early Aβ deposition and key features of AD. We assessed the effects of DPIN on hippocampal, intestinal, and plasma markers, including Aβ, tau, CDK5, the PI3K/Akt pathway, gut microbiota composition, behavioral changes, blood–brain barrier integrity, and systemic inflammation. This comprehensive approach aims to provide novel insights into DPIN’s disease-modifying potential in AD.

## 2. Materials and Methods

### 2.1. Animals and Ethics Statement

Animals used in this experiment were non-transgenic (Non-Tg) and homozygous (5×FAD) male and female mice. The transgenic 5×FAD mice co-express and co-inherit familial Alzheimer’s disease (FAD) mutant forms of the sshuman amyloid precursor protein gene (hAPP) (the Swedish mutation: K670N, M671L; the Florida mutation: 1716V; the London mutation: V7171) and presenilin-1 gene (PSEN-1) (M146L and L286V mutations). Transgenes under transcriptional control of the neuron-specific mouse Thy-1 promoter (Tg6799 line) [48,49]. 5×FAD lines (B6/SJL genetic background) were maintained by crossing heterozygous transgenic mice with B6/SJL F1 breeders (The Jackson Laboratory, Bar Harbor, ME, USA). Wild-type Non-Tg littermate mice served as controls. The rodents were housed individually in the Animal Centre for Experimentation at the University of Malaga with water and food provided ad libitum under standardized conditions: a 12 h light/dark cycle, 20 ± 2 °C of room temperature, and 40 ± 5% of relative humidity.

All experiments were realized in compliance with the ARRIVE guidelines [50] and concordance with the European Communities Council Directives 2010/63/EU, Regulation (EC) n° 86/609/ECC (24 November 1986), and Spanish National and Regional Guidelines for Animal Experimentation (Real Decreto 53/2013). Experimental protocols were approved by The Local Ethical Committee for Animal Research of the University of Malaga (CEUMA n° 203-2023-A, 1 April 2024). Accordingly, all efforts were made to minimize animal suffering and to reduce the number of animals used. Potential confounders, such as the order of treatments and measurements, and animal/cage location, were minimized by standardizing the timing of treatments and measurements for all animals and ensuring consistent environmental conditions across cages. Additionally, cage locations were rotated periodically to prevent location bias. These measures were implemented to reduce the influence of external variables on the study outcomes.

### 2.2. Pharmacological Treatment with Pure D-Pinitol

**Figure 1:**
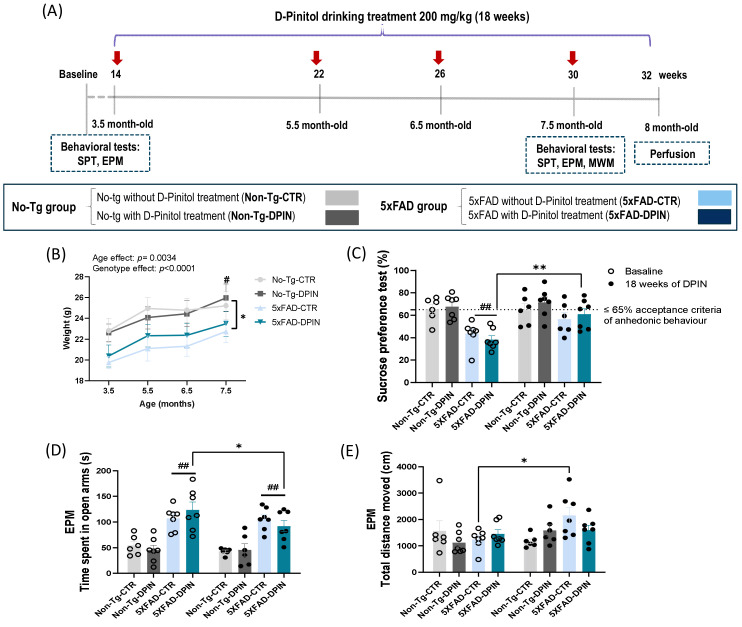
Anhedonic and anxiety-like behavior after 18 weeks of D-Pinitol treatment in 5×FAD mice. (**A**) Experimental procedure where mice were supplemented with D-Pinitol (DPIN, 200 mg/kg/day) ad libitum in their drinking water for 18 weeks. All animals were about 14 weeks of age (3.5 months old) at the beginning of the experiment. Experimental groups: non-transgenic (Non-Tg-DPIN; n = 16; 7 males; 9 females) and transgenic 5×FAD (5×FAD-DPIN; n = 17; 10 males; 7 females) mice. Control groups of both genotypes: non-transgenic (Non-Tg-CTR; n = 16; 7 males; 9 females), and 5×FAD transgenic mice (5×FAD-CTR; n = 14; 7 males; 7 females) received water as a vehicle solution. Animal control weight (CW) was recorded at 3.5–5.5–6.5–7.5 months of age). Behavioral tests were performed at baseline point (3.5 months old) and after 16 weeks with DPIN treatment (8 month old): sucrose preference test (SPT) and elevated plus maze (EPM). The Morris water maze (MWM) behavioral test began at 7.5 months of age and was finalized at 8 months of age. The animals were sacrificed at 32 weeks of age (8 months) and tissue samples were rapidly removed. (**B**) Body weight in grams (g). Two-way ANOVA test: (*) *p*< 0.05 genotype effect; (#) *p*< 0.05 age effect. (**C**) Sucrose preference test (%) at the baseline point and after 18 weeks of DPIN treatment. Dashed lines represent the criterion for anhedonia ≤ 65%. Two-way ANOVA and Tukey’s test: (##) *p* < 0.01 between 5×FAD (5×FAD-CTR and 5×FAD-DPIN) compared to Non-Tg mice (Non-Tg-CTR and Non-Tg-DPIN) at the baseline point. (**D**) Time spent in seconds (s) in the open arms at the baseline point and after 18 weeks of DPIN treatment in the EPM. Two-way ANOVA and Tukey’s test: (##) *p* < 0.01 between 5×FAD (5×FAD-CTR and 5×FAD-DPIN) compared to Non-Tg mice (Non-Tg-CTR and Non-Tg-DPIN) at the baseline point and after 18 weeks of DPIN. (**E**) Total distance moved in centimeters (cm) at the baseline point and after 18 weeks of DPIN treatment in the EPM test. Results are shown as the mean ± SEM. Two-way ANOVA and Tukey’s test from (**C**–**E**): (*) *p* < 0.05 and (**) *p* < 0.01 in the 5×FAD mice after 18 weeks of DPIN treatment.

Mice were fed with a standard pellet diet (3.02 kcal/g with 30 kcal% protein, 55 kcal% carbohydrates, and 3015 kcal% fat; Harlam; Tecklad, Madison WI, USA) supplemented with D-Pinitol (3-O-methyl-D-chiro-inositol, Caromax^®^-D-Pinitol, DPIN, 98% purity; Euronutra S.L., Málaga, Spain) ad libitum in their drinking water for 18 weeks (4.5 months). All animals were about 14 weeks of age (3.5 months old) at the beginning of the experiment. Daily doses were 200 mg/kg/day, as a dissolved powder in water bottles (1 mg/mL, consuming 5 mL/day per 25 mg of body weight). Over the 18-week treatment period, the water bottles containing D-Pinitol were replaced daily, from Monday to Sunday. This daily replacement allowed us to control the amount consumed by each mouse. We established the following experimental groups: non-transgenic (Non-Tg-DPIN; n = 16; 7 males; 9 females) and transgenic 5×FAD (5×FAD-DPIN; n = 17; 10 males; 7 females) mice that received DPIN as treatment. Control groups of both genotypes were non-transgenic (Non-Tg-CTR; n = 16; 7 males; 9 females), and 5×FAD transgenic mice (5×FAD-CTR; n = 14; 7 males; 7 females) received water as a vehicle solution. The sample size was determined based on resource availability and existing literature on similar studies. We controlled the weight of the animals during the experiment (Figure 1A). Animal weight was recorded at 3.5–5.5–6.5–7.5 months of age, the period in which the treatment of DPIN was administered. Finalizing the DPIN administration for 18 weeks, the animals were anesthetized at 32 weeks of age (8 months old) with intraperitoneal sodium pentobarbital injection (50 mg kg^−1^ BW) 5 min before the mice were sacrificed. Tissue samples were rapidly removed and immediately frozen at −80 °C for later analysis (refer to Section 2.4), taking the whole process less than 10 min from the administration of pentobarbital.

### 2.3. Behavioral Testing

#### 2.3.1. General Considerations for Behavioral Assessment

Three days before the first behavioral test, animals were habituated to handling. On each testing day, mice were allowed to adapt in a behavior room for at least 30 min. The illumination of the arena or maze was 35–40 lux. Video tracking software recorded and assessed the test (Ethovision XT 12.0, Noldus, Wageningen, The Netherlands).

#### 2.3.2. Sucrose Preference Tests

To analyze anhedonia in our mouse model of AD, we carried out the sucrose preference tests (SPT). Mice were trained to consume sucrose for 24 h for 2 days before the start of the test. For the preference test, animals were free to choose between 2 pre-weighed bottles for 24 h, one containing tap water and one containing 0.05% sucrose (Sigma-Aldrich, St. Louis, MO, USA). The position of bottles was counterbalanced among animals to prevent possible effects of side preferences in drinking behavior. The animals’ consumption was measured by weighing the bottles. SPT was calculated according to the following formula: (sweet solution intake)/(sweet solution intake + water intake) × 100 [51]. Criterion acceptance of preference was ≤65%; below this value was considered anhedonia [51]. Weighing of all the mice occurred for 4 days, including the first day before training, followed by the next two days during training, and finally, on the fourth day during the final test.

#### 2.3.3. Elevated Plus Maze (EPM)

Anxiety-like behavior was evaluated through the elevated plus maze [52,53]. The maze consisted of two open arms (30 × 5 cm), two enclosed arms (30 × 5 × 15 cm), and a connecting central platform (5 × 5 cm). The maze was raised to a height of 38.5 cm above the floor. The mouse was placed in the center of the EPM, and its behavior was recorded for 5 min. We assessed the following behavior: locomotion (distance moved), the time spent in the open arm, and the number of entries into the arms.

#### 2.3.4. Morris Water Maze (MWM)

The Morris water maze was conducted to study learning and memory, following procedures already described [54]. The maze consisted of a circular pool (120 cm diameter) filled with nontoxic opaque water at 23 ± 2 °C temperature and divided into four equal quadrants (Q1–Q4). The escape platform (10 cm diameter) was raised 1 cm above the water surface for cue visual training. The hidden platform was located 0.5 cm below the water surface for the acquisition and reversal of spatial learning. The room testing was decorated with eye-catching visual cues to aid the mice in orienting themselves concerning the pool. We examined the following learning parameters: escape latency, path length (total distance navigated), swimming speed, and cumulative distance to the platform as a measure of spatial learning ability [55] and the time spent on the quadrants. See Supplementary Information (SI) Figure S1A for more details of the learning protocol followed.

### 2.4. Tissue and Histological Procedures

#### 2.4.1. Sample Collection

Before sacrifice, blood was drawn directly from the right atrium. It was centrifuged (2100× *g* for 10 min) for plasma collection, and it was kept at −80 °C for biochemical analysis. After that, animals were transcranially perfused with 0.1 M phosphate-buffered saline (PBS). Next, colon stools were collected and frozen immediately at −80 °C for microbiota analysis. Brain samples were rapidly removed and bisected down the midline; the left hemi-brain was stored in 4% paraformaldehyde (PFA) for histological procedures, and the right hemi-brain was kept in dry ice for storage at −80 °C for biochemical analysis. Small intestine samples were also collected and stored in 4% PFA and dry ice at −80 °C for histological and biochemical analysis, respectively.

#### 2.4.2. Immunohistochemistry and Immunofluorescence

Regarding Aβ plaque immunohistochemistry in the brain, the hemi-brains were post-fixed in 0.1M PBS containing 4% PFA for 48 h and cryopreserved in 30% sucrose in 0.1 M PBS solution for 5 days at 4 °C. Hemi-brains were cut at a 50 µm thickness in the coronal plane on a microtome. Serial sections were blocked with 5% donkey serum and 0.5% Triton X-100 in 0.1 M PBS for 45 min at room temperature (RT). For analysis of amyloid-β plaques, we used rabbit anti-Aβ1-42 (Aβ42; 1:500, ThermoFisher, Waltham, MA, USA) and rabbit anti-Aβ1-40 (Aβ40; 1:200, ThermoFisher). Primary antibodies were incubated overnight at RT. After rinsing, the sections were incubated with the secondary antibody biotinylated goat anti-rabbit (1:800, GE Healthcare, Chicago, IL, USA) for 2 h at RT. All antibodies were diluted in PBS, 0.5% Triton X-100, and 2.5% donkey serum. We used the peroxidase-conjugated ExtraAvidin method and diaminobenzidine as the chromogen to visualize the reaction product.

Images were acquired with a digital camera DP70 (Olympus Iberia, S.A.U., Barcelona, Spain) connected to an Olympus BX41 microscope, binarized to 16-bit black and white, and a fixed intensity threshold was applied for each immunostaining. The number of labelled Aβ plaques was manually counted across the entire slices, including the CA1, CA2, and dentate gyrus (DG) regions, using ImageJ software Version 1.53f51 [56]. Eight mice per group and three sections per mouse were used for hippocampal quantification.

#### 2.4.3. Western Blot Analysis

Western blotting was performed as described previously [41]. Briefly, frozen hemi-brain samples (17 mg per sample) were dissected and homogenized in 1 mL of cold rssadioimmunoprecipitation assay buffer lysis buffer (RIPA); 50 mM Tris-HCl pH 7.4, 150 mM NaCl, 0.5% NaDOC, 1 mM EDTA, 1% Triton, 0.1% SDS, 1 mM Na_3_VO_4_, 1 mM NaF, supplemented with a protease (cOmpleteTM Protease Inhibitor Cocktail, Roche, cat. number 11836145001, Basel, Switzerland) and a phosphatase (Phosphatase Inhibitor Cocktail Set III, Millipore, cat. number 524527, Darmstadt, Germany) inhibitor cocktail. The suspension was incubated for 2 h at 4 °C, followed by centrifugation at 12,000 rpm for 15 min at 4 °C. The supernatant was transferred to a new clean centrifuge tube, and the Bradford colorimetric method was used to determine the concentration of the total protein. The protein extracts were diluted 1:1 in the loading buffer (Dithiothreitol (DTT) 2X) and heated for 5 min at 99 °C before being subjected to electrophoresis.

The tissue protein (10–15 μg) was subjected to electrophoresis on 4–12% Criterion XT Precast Bis-Tris gels (Bio-Rad, Hercules, CA, USA, EE.UU.) for 30 min at 80 V and 2 h at 150 V. Proteins were transferred onto a 0.2 µm nitrocellulose membrane (Bio-Rad, Hercules, CA, USA, EE.UU.) for 1 h at 80 V by wet transfer equipment. The membrane was washed twice for 5 min in TBST (10 mM Tris–HCl, 150 mM NaCl, 0.1% Tween 20, pH 7.6) and blocked with 5% BSA-TBST for one hour at RT on a shaker platform. Subsequently, the membrane was incubated with the respective primary antibodies overnight at 4 °C diluted in 2% BSA-TBST (see Supplementary Information (SI) Table S1 for additional information regarding primary antibodies). On the next day of primary antibody incubation, the membrane was washed three times for 5 min with TBST. An appropriate HRP conjugated rabbit/mouse secondary antibody (Promega, Madison, WI, USA, EE.UU.) was diluted 1:10,000 in 2% BSA-TST and incubated with the membrane for 1 h shaking at room temperature. Finally, the membrane was washed as above and exposed to chemiluminescent reagent using a Western Blotting Luminol Reagent kit (Santa Cruz Biotechnology, Santa Cruz, CA, USA) for 1 min. The membrane-bound protein was then visualized by chemiluminescence using the Chemi-Doc TM MP Imaging System (Bio-Rad, Barcelona, Spain). After detecting phosphorylation proteins, the particular antibodies were removed from the membrane by incubating with stripping buffer (2% SDS, 62.5 mM Tris HCl (pH 6.8), 0.8% β-mercaptoethanol) for 30 min at 50 °C (see Supplementary Information (SI) Figures S2 and S3 for additional information regarding the stripping process). Membranes were thoroughly cleaned in ultrapure water before being pre-incubated with the matching total antibody. Bands were quantified by densitometry analysis using ImageJ software Version 1.53f51 (https://imagej.net/ij/, accessed on 1 January 2024). Normalization was performed using the reference protein γ-Adaptin of the same membrane. The results are presented as the ratio between total protein expression and γ-Adaptin, and the ratio between phosphorylated protein expression and total protein expression [57].

#### 2.4.4. RT-qPCR

We performed real-time PCR as described previously [58]. Briefly, total RNA was extracted from tissue sections of the small intestine (50–80 mg) using the Trizol^®^ method, according to the manufacturer’s instructions (Invitrogen, Carlsbad, CA, USA). RNA samples were isolated with an RNA easy minelute cleanup kit, including digestion with DNase I column (Qiagen, Hilden, Germany), and quantified using a spectrophotometer to ensure A260/280 ratios of 1.8–2.0. Reverse transcription was carried out from 1 ug of RNA using the Transcriptor Reverse Transcriptase kit (Transcriptor RT, Roche Applied Science, Mannheim, Germany) and specific sets of primer probes (claudin-3: Cldn3, Mm00515499_s1, amplicon length: 60; occludin: Ocln, Mm00500910_m1, amplicon length: 83; TLR4: Tlr4, Mm00445273_m1; amplicon length: 87) from TaqMan^®^ Gene expression Assays (TaqMan, ThermoFisher Scientific, Waltham, MA, USA). Real-time qPCR reactions were carried out in a CFX96TM Real-Time PCR Detection System (Bio-Rad, Hercules, CA, USA) and the FAM dye labelled format for the TaqMan^®^ Gene Expression Assays (ThermoFisher Scientific). A melting curve analysis was performed to ensure that only a single product was amplified. We normalized values obtained from the small intestine samples concerning Gapdh levels (Mm99999915_g1, amplicon length: 107; ThermoFisher Scientific).

### 2.5. Measurement of Plasma Metabolic and Inflammatory-Related Indicators by Multiplex and ELISA

Insulin, leptin, ghrelin, glucagon, and plasminogen activator inhibitor-1 (PAI-1) plasma levels were measured using a multiplex immunoassay system with the commercial kit: Bio-Plex Pro™ mouse diabetes 8-plex immunoassay, cat. number #171F7001M (Bio-Rad, Hercules, CA, USA). Plates were run on a Bio-Plex MAGPIX™ Multiplex Reader (Luminex, Austin, TX, USA) with Bio-Plex Manager™ MP Software (https://www.bio-rad.com/en-rs/product/bio-plex-manager-software-standard-edition, accessed on 1 January 2024). Hormone concentrations were expressed in pg/mL, and detection limits were 68.29 (insulin), 5.07 (leptin), 0.64 (ghrelin), 0.50 (glucagon), and 2.98 (PAI-1) pg/mL.

Plasma levels of the proinflammatory cytokines Interleukin 5 (IL-5), Interleukin 6 (IL-6), Keratinocyte chemoattractant (KC)/human growth-regulated oncogene (GRO), and Tumor necrosis factor alpha (TNF-α) were measured using a multiplex immunoassay system with V-PLEX Validated Assay Kits: MSD cytokine assay, proinflammatory panel 1 (mouse) kit, cat. number #K15048D-1 (Meso Scale Diagnostics, Rockville, MD, USA). Cytokine concentrations were expressed in pg/mL, 2-fold dilution. The plate was analyzed on an MSD instrument.

Plasma LPS in mice was determined according to the corresponding ELISA kit and accompanying operating instructions, cat. number #CSB-E13066m (Cusabio Technology LLC, Houston, TX, USA).

### 2.6. Gut Microbiota Analysis

Analysis of gut microbiota was performed as previously described [59]. Colon feces samples (180–200 mg) were used for DNA extraction. The QIAamp^®^ DNA Stool Mini Kit (Qiagen France S.A.S., Rennes, France) was used according to the manufacturer’s instructions. DNA concentration and purity were determined by absorbance at 260 nm (A260) and A260/A280 ratio, respectively, using a NanoDrop spectrophotometer (NanoDrop TM One Spectrophotometer, Thermo Fisher Scientific Inc., Waltham, MA, USA). DNA samples were sent to StarSEQ^®^ GmbH (Mainz, Germany) for the analysis of the V3–V4 hypervariable regions of the bacterial 16 S rRNA gene, amplified from the isolated DNA using the primer combination 515F–909 R. The Illumina MiSeq System was used to sequence DNA products of this PCR amplification, and the 16 S metagenomics analysis was performed using the QIIME2 [60] version 2023.5 in a Conda environment, which is based on the Ubuntu Linux operating system. The taxonomy database used was Silva [61] 138.1 version, and the RESCRIPt plugin [62] was employed for its curation and preparation. Phylum analysis focused on both the Bacteroidetes and Firmicutes phyla. Taxonomic compositions were compared at the family level in terms of relative abundance using QIIME 2.

### 2.7. Statistical Analyses

All data are expressed as mean ± standard error of the mean (SEM). Statistical analysis for behavioral tests, Western blot, PCR, immunostaining quantification, ELISA, and multiplex analysis was conducted in GraphPad Prism, version 9 (GraphPad Software, Inc., La Jolla, CA, USA). The Shapiro–Wilk test was used to assess the normal distribution of data. Levene’s test was used to analyze the assumption of homogeneity of variance. Statistical analysis was undertaken for studies where each group size was, at least, n = 5. One- and two-way analysis of variance (ANOVA) was assessed, followed by Turkey’s post hoc multiple comparisons test. The post hoc tests were conducted only if F in one-way ANOVA or the interaction between factors in two-way ANOVA tests achieved a *p*-value less than 0.05 and there was no statistically significant variance inhomogeneity. The results were considered statistically significant at *p* ≤ 0.05. Non-significant (ns) results will be above 0.05. Details of the statistical analyses performed are provided in the Supplementary Information section, under “Statistical Analyses”.

For microbiota analysis, sequencing data was processed and analyzed using specific software and packages. The paired-end sequences of each sample were exported in FASTQ format. The quality of the sequences was first assessed using the FastQC software, Version 0.12.0. For the quantitative analysis of the relative abundance of the microbiota in the samples, R 4.1.2 was used in the RStudio working environment, version 2023.03.1. The analysis utilized the “stats”, “xlsx”, “car”, “dplyr”, and “readr” packages, along with the Bioconductor “bio format” package. A paired-end demultiplexed sequencing protocol was used to import the sequences, and the dada2 denoise-paired command was employed for quality filtering, denoising, and merging of paired-end reads. Core metrics were obtained to assess alpha and beta diversities. Alpha diversity measures the species richness within each community, while beta diversity examines the differences in composition, specifically the abundance of different taxa, among different samples. Alpha rarefaction analysis was performed at a depth of 25,000 per sample. The sequences were grouped into operational taxonomic units (OTUs) using a 97% similarity threshold. Taxonomic assignment was carried out using a trained classifier using the q2-feature-classifier plugin. Statistical inference was performed using the Kruskal–Wallis test and Mann–Whitney U test for each OTU, allowing for comparisons and identification of significant differences between groups.

To address the potential issue of Type-1 error arising from multiple outcome measures, power analysis and sample size estimation were performed before the initiation of the study. Effect sizes were estimated based on previous literature to ensure adequate statistical power for the multitude of outcome measures. Additionally, adjustments for multiple comparisons were considered in our statistical analyses to enhance the reliability of the findings.

## 3. Results

### 3.1. D-Pinitol Treatment Ameliorates Anhedonic and Abnormal Emotional Behavior in the 5×FAD Mouse Model

Animal weight during the test was recorded at 3.5–5.5–6.5–7.5 months of age, the period in which the treatment of DPIN was administered. The two-way ANOVA test revealed a ‘genotype’ and ‘age’ effect (Figure 1B). Specifically, Non-Tg mice displayed a more pronounced age-dependent weight gain compared to 5×FAD. Importantly, D-Pinitol treatment did not significantly affect body weight across any of the time points analyzed. The baseline difference likely reflects inherent variability between groups rather than an effect of treatment, as 5×FAD mice have been reported to exhibit altered weight dynamics due to their genotype. Therefore, our findings indicate that both genetics and age play a role in body weight. D-Pinitol, however, did not appear to affect body weight in this study.

Regarding the SPT test, two-way ANOVA showed a genotype effect on SPT since sucrose intake was significantly lower in the 5×FAD compared to Non-Tg mice at the baseline point (two-way ANOVA; ‘genotype’ effect, *p* < 0.01; Figure 1C). Post hoc analysis showed that after 18 weeks of treatment, the 5×FAD group consuming DPIN significantly increased sucrose intake, almost reaching the anhedonic threshold set at 65% (two-way ANOVA; Tukey’s test; *p* < 0.01; Figure 1C). In contrast, although there is a trend towards higher sucrose consumption due to age in the 5×FAD control group, the preference is still low and not significant.

An EPM test was conducted to assess emotional status by monitoring anxiety-like behaviors. In EPM, spending less time in open arms has been associated with anxiety-like behavior [63]. Two-way ANOVA revealed a genotype effect regarding the time spent in the open arms (two-way ANOVA; ‘genotype’ effect, *p* < 0.0001; Figure 1D). These results indicate abnormal emotional behavior in the 5×FAD compared to Non-Tg mice, as previously described [54,64]. Moreover, post hoc analysis showed that the 5×FAD mice after DPIN treatment significantly reduced time in open arms (Figure 1D). Differences in locomotion can be seen in the 5×FAD control with age, traveling significantly more total distance (time in open and closed arms) (Figure 1E). In contrast, treatment with DPIN slows down the distance travelled.

### 3.2. D-Pinitol Treatment Improves Cognitive Function in the Mouse 5×FAD Evaluated in the Morris Water Maze

**Figure 2:**
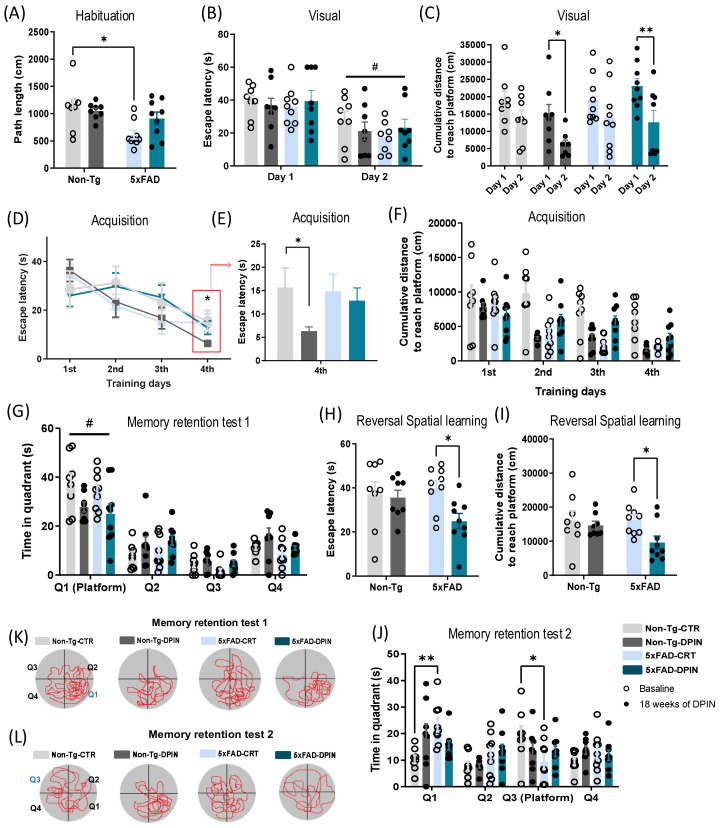
Assessment of cognitive function by Morris water maze after 18 weeks of D-Pinitol treatment. Data is presented as the mean ± SEM. Two-way ANOVA + Tukey’s test for multiple comparisons were performed. (**A**) Path length in centimeters (cm) (* *p* < 0.05) during the habituation training. (**B**) During the visual training (2 days, visible platform; 4 trials/day), all experimental groups diminished the escape latency (s) on the second day (# *p* < 0.05 day 2 vs. day 1). (**C**) Non-Tg-DPIN and 5×FAD-DPIN showed a reduced cumulative distance (cm) to reach the platform on the second training day (* *p* < 0.05 and ** *p* < 0.01). (**D**) On acquisition training, each subject received six trials per acquisition day (4 days, hidden platform; 6 trials/day). The escape latency (s) was decreased on the last and the fourth training day (* *p* < 0.05), (**E**) being more significant in the Non-Tg-DPIN experimental group (* *p* < 0.05). (**F**) The cumulative distance (cm) to reach the hidden platform was also evaluated and showed a similar profile to the escape latency outcomes on acquisition training. (**G**) On memory retention test 1 (without platform; 1 trial/day), all animals demonstrated similar measures of time (s) spent searching the target quadrant (Q1) (# *p* < 0.05 Q1 vs. the other quadrants). (**H**) After 48 h, each subject received six trials for one day on the reversal spatial learning day (1 day, hidden platform; 6 trials/day). 5×FAD-DPIN reached the new hidden platform position significantly faster (s) than 5×FAD-CTR (* *p* < 0.05) and (**I**) with less distance traveled (cm) (* *p* < 0.05). (**J**) On memory retention test 2, 5×FAD-CTR exhibited impaired long-term spatial memory as measured by less time spent (s) in the new position of the platform (Q3) and persisted for a longer period on the Q1 position that they learned on the acquisition training compared to Non-Tg-CTR (* *p* < 0.05, ** *p* < 0.01). (**K**,**L**) shows a graphical representation of the path traveled by each group during the first and second memory retention tests.

After 18 weeks of continuous drinking treatment with DPIN, we analyzed learning and memory through the MWM. On the habituation training, a two-way ANOVA was conducted on path length and swimming speed as well as each zone (periphery, intermediate, and center zones). Genotype and treatment factors were considered. While no interaction or treatment factor showed significant results, there was a ‘genotype’ effect (two-way ANOVA; ‘genotype’ effect, *p* < 0.01; Figure 2A) on path length. Although transgenic 5×FAD mice had a shorter path length than the non-transgenic control group, there was no effect on swimming speed (Supplementary Information (SI) Figure S1B). All mice swam around the different zones of the pool with a similar swimming speed regardless of DPIN treatment and genotype (Figure S1B). However, all groups showed a significantly longer path length and swimming speed in the peripheral zone than in the central zone, showing a positive thigmotactic behavior (two-way ANOVA; ‘zones’ effect, *p* < 0.0001; Supplementary Figure S1C,D). Regarding the path length, we also noticed a significant decrease in distance travelled at the peripheral zone between Non-Tg-CTR and 5×FAD-CTR (Supplementary Information (SI) Figure S1D).

Concerning visual training, two-way ANOVA repeated measures showed a ‘genotype × treatment’ effect on escape latency comparing both days (two-way ANOVA; ‘genotype × treatment’ effect, *p* < 0.0001; Figure 2B). Post hoc comparison showed that all four groups reached the visible platform faster on the second day of training (Figure 2B). The cumulative distance to reach the platform was different between groups, having a ‘trial session [day]’ and a ‘genotype × treatment’ effect (two-way ANOVA; ‘trial session [day]’ effect, *p* < 0.05; ‘genotype × treatment’ effect, *p* < 0.001; Figure 2C). Post hoc comparison revealed that only Non-Tg-DPIN and 5×FAD-DPIN groups significantly reduced the cumulative distance to reach the visible platform on the second training day (Figure 2C), showing acquired learning.

On acquisition training, two-way ANOVA repeated measures revealed a ‘trial session [day]’ effect on escape latency (two-way ANOVA; ‘trial session [day]’ effect, *p* < 0.0001; Figure 2D). On the fourth and last acquisition training day, surprisingly the Non-Tg-DPIN group showed reduced escape latency values compared to the rest of the groups (*t*-Test, *p* < 0.05; Figure 2E), while the 5×FAD-DPIN group exhibited values similar to 5×FAD-CTR. The results about the cumulative distance to reach the hidden platform matched the escape latency ones, showing a ‘trial session [day]’ and a ‘genotype × treatment’ effect (two-way ANOVA; ‘trial session [day]’ effect, *p*< 0.0001; ‘genotype × treatment’ effect, *p* < 0.0001; Figure 2F). After twenty hours, in the first memory retention test, all mice spent more time on the target quadrant (Q1) (two-way ANOVA; ‘quadrant’ effect, *p* < 0.0001; Figure 2G) as well as higher path length on it (two-way ANOVA; ‘quadrant’ effect, *p* < 0.0001; (Supplementary Information (SI) Figure S1E), with no significant differences between DPIN and control groups.

On reversal spatial learning, two-way ANOVA showed a ‘treatment’ effect on escape latency (two-way ANOVA; ‘treatment’ effect, *p* < 0.05; Figure 2H) and cumulative distance (two-way ANOVA; ‘treatment’ effect, *p* < 0.05; Figure 2I). 5×FAD mice receiving DPIN reached the new hidden platform position significantly faster than 5×FAD-CTR, demonstrating a lower escape latency compared to its control. After twenty hours, 5×FAD-CTR demonstrated impaired long-term spatial memory due to a change in the platform’s position on the second memory retention test. 5×FAD-CTR mice showed less time spent searching in the correct position (Q3) and persisted for a longer period on the Q1 that they learned on the acquisition training compared to Non-Tg-CTR (two-way ANOVA; ‘quadrant’ effect, *p* < 0.001; Figure 2J). However, 5×FAD mice treated with DPIN spent a similar time in Q1 and Q3 without persisting for so long in Q1, showing that DPIN treatment ameliorates cognitive spatial flexibility. Regarding the path length, no differences between groups were seen (Supplementary Information (SI) Figure S1F). Figure 2K,L show a graphical representation of the path travelled by each experimental group during the first and second memory retention tests.

### 3.3. Regulation of Hormones Related to Insulin and Metabolic Health in Alzheimer’s Disease Transgenic Mice After D-Pinitol Treatment

**Figure 3:**
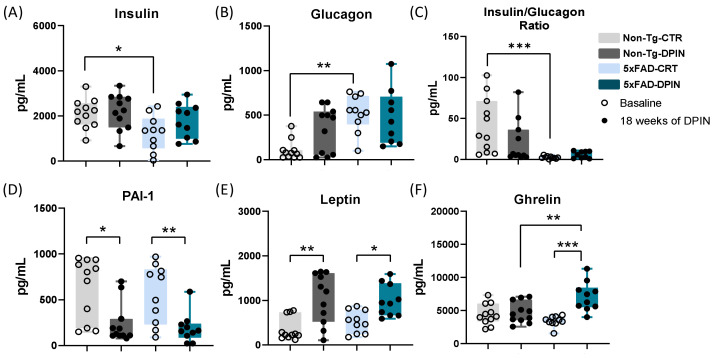
Regulation of hormones related to insulin and metabolic health after 18 weeks of D-Pinitol treatment. Plasma levels (pg/mL) of (**A**) insulin, (**B**) glucagon, (**C**) insulin/glucagon ratio, (**D**) plasminogen activator inhibitor-1 (PAI-1), (**E**) leptin, and (**F**) ghrelin. Histograms represent mean ± SEM (n = 10). Two-way ANOVA and Tukey’s test for multiple comparisons were performed: (*) *p* < 0.05, (**) *p* < 0.01, and (***) *p* < 0.001.

Next, we evaluated insulin-linked metabolic pathways by monitoring plasma levels of peptides and hormones involved in modulating insulin release and signaling. Regarding the effects of DPIN on insulin (one-way ANOVA: *p* < 0.05; Figure 3A), while the 5×FAD-CTR group showed a significant drop compared to the Non-Tg-CTR group, DPIN was able to restore insulin levels in the transgenic group. This suggests that DPIN may be beneficial for improving glycemic regulation in AD. As for glucagon (one-way ANOVA: *p* < 0.001; Figure 3B), which is a hormone that opposes the effects of insulin, the results show that levels are significantly higher in the transgenic control group. However, the 5×FAD treated group does not show differences if compared with the Non-Tg mice as seen before with the insulin level. The insulin/glucagon ratio has an inverse correlation with liver glucose production. A high ratio suggests that glucose is being stored and there is an increase in protein synthesis. On the other hand, a low ratio indicates an increase in gluconeogenesis, which is the process of producing glucose from amino acids. This ratio was significantly lower in the 5×FAD-CTR group compared to the Non-Tg group (one-way ANOVA: *p* < 0.001; Figure 3C). As for plasminogen activator inhibitor-1 (PAI-1) (one-way ANOVA: *p* < 0.001; Figure 3D), the results showed that DPIN decreases PAI-1 levels in both the transgenic and Non-Tg groups. Regarding leptin and ghrelin, hormones involved in appetite, satiety regulation, and body weight regulation, the results showed a notable rise in leptin levels in both groups with DPIN (one-way ANOVA: *p* < 0.001; Figure 3E) and a significant increase in ghrelin levels in the transgenic group with DPIN (one-way ANOVA: *p* < 0.0001; Figure 3F).

### 3.4. Activation of the PI3K/Akt Pathway in the Hippocampus of 5×FAD Mice After 18 Weeks of Continuous D-Pinitol Treatment

**Figure 4:**
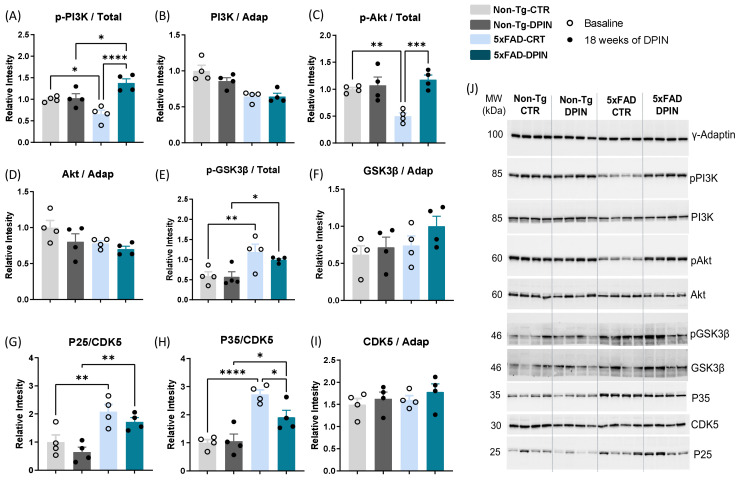
Activation of the hippocampal PI3K/Akt pathway after 18 weeks of D-Pinitol treatment. Western blot analysis of the phosphorylation status of the p85 regulatory domain of (**A**) the phosphatidylinositol 3 kinase (p85-PI3K) phosphorylation at tyrosine 607, (**B**) and the quantity of total p85-PI3K, (**C**) protein Kinase B (Akt) phosphorylation on serine 473, (**D**) and the amount of total Akt, (**E**) glycogen synthase kinase 3β (GSK-3β) phosphorylation at serine 9, (**F**) and the amount of total GSK-3β, (**G**) cyclin-dependent kinase 5 (CDK5) subunits p25 (**H**) and p35, (**I**) and the total quantity of CDK5 on Non-Tg and 5×FAD with (DPIN) and without (controls = CTR) D-Pinitol treatment. (**J**) The blots represent all bands. Molecular weights (MWs) are expressed in kilodaltons (kDa). The corresponding expression of γ-Adaptin is shown as a loading control per lane. All samples were obtained simultaneously and processed in parallel. Histograms represent mean ± SEM (n = 4). Two-way ANOVA and Tukey’s test for multiple comparisons were performed: (*) *p* < 0.05, (**) *p* < 0.01, (***) *p* < 0.001 and (****) *p* < 0.0001.

After 18 weeks of oral DPIN treatment, we observed an activation of the PI3K/Akt pathway. First, we examined the phosphorylation state of PI3K (p-PI3K), which was significantly lower in 5×FAD mice compared to in Non-Tg animals (one-way ANOVA: *p* < 0.001; Figure 4A). However, the 5×FAD-DPIN group showed a significant increase in PI3K phosphorylation status after DPIN treatment (one-way ANOVA: *p* < 0.001; Figure 4A), while the levels of total PI3K protein remained unchanged regardless of genotype and treatment (one-way ANOVA: *p* = ns; Figure 4B). Subsequently, we analyzed the phosphorylation state of Akt (p-Akt), showing that DPIN treatment reversed the low phosphorylation observed in the 5×FAD control animals (one-way ANOVA: *p* < 0.05; Figure 4C), while the levels of total Akt protein remained unchanged (one-way ANOVA: *p* = ns; Figure 4D). According to increased activity of Akt, the phosphorylation in serin 9 (Ser9) of its substrate GSK3β (p-GSK3β) was also increased after DPIN treatment in the 5×FAD mice (one-way ANOVA: *p* < 0.05; Figure 4E), thus inhibiting GSK3β activity, with no changes in the total amount of GSK3β (one-way ANOVA: *p* = ns; Figure 4F).

CDK5 (cyclin-dependent kinase 5) is a protein kinase that has been found to interact with several components of the PI3K/Akt signaling pathway. CDK5 acts by interacting with cyclin-dependent kinase 5 regulatory subunit 1 (CDK5R1), a protein also known as P35 and a cell-periphery activator of CDK5. Cleavage of p35 by calpain, or other proteolytic enzymes, releases a fragment, P25, that permanently activates CDK5. In this study, we observed that 5×FAD mice had higher levels of P25 protein regardless of treatment (one-way ANOVA: *p* < 0.01; Figure 4G). When we examined the P35 protein, its levels were higher in 5×FAD compared to Non-Tg animals. However, in the 5×FAD group, treatment with DPIN for 18 weeks resulted in a significant decrease in the P35/CDK5 ratio (one-way ANOVA: *p* < 0.01; Figure 4H). The total protein levels of CDK5 did not show significant changes in either genotype or treatment groups (one-way ANOVA: *p* = ns; Figure 4I).

### 3.5. D-Pinitol Reduces Key Protein Markers of Alzheimer’s Disease

**Figure 5:**
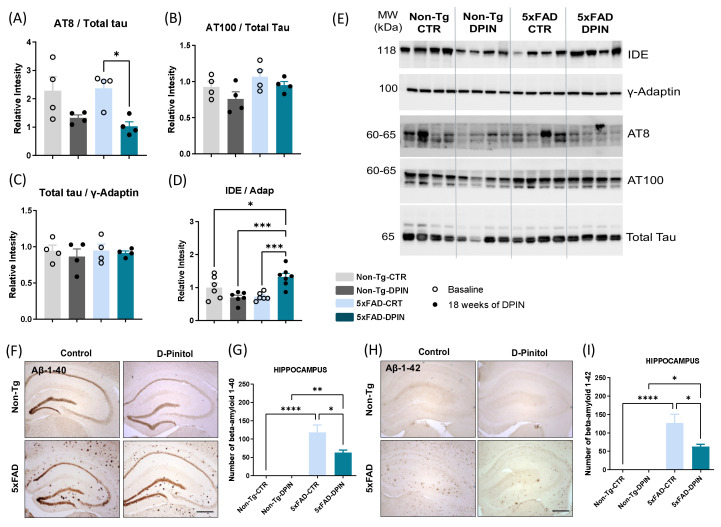
Amyloid beta clearance and tau dephosphorylation in the hippocampus of 5×FAD mice after 18 weeks of D-Pinitol treatment. Western blot analysis of (**A**) tau [AT8] phosphorylation on serine 202 and threonine 205, (**B**) tau [AT100] phosphorylation on threonine 212 and serine 214, (**C**) the total amount of tau, and (**D**) insulin-degrading enzyme (IDE) on Non-Tg and 5×FAD with (DPIN) and without (controls = CTR) D-Pinitol treatment. (**E**) The blots represent all bands. Molecular weights (MW) are expressed in kilodaltons (kDa). The corresponding expression of γ-Adaptin is shown as a loading control per lane. All samples were obtained simultaneously and processed in parallel. Histograms (**A**–**D**) represent mean ± SEM (n = 4). (**F**,**H**) Images correspond to representative immunostaining of Aβ 1-40 (Aβ 1-40) and Aβ 1-42 (Aβ 1-42) densitometry in the hippocampus of Non-Tg and 5×FAD controls (CTR) and after 18 weeks of continuous drinking treatment with D-Pinitol (DPIN). Scale bar: 100 µm. Histograms in (**G**,**I**) represent the mean ± SEM of the number of Aβ from all samples per group (n = 8). Two-way ANOVA and Tukey’s test for multiple comparisons were performed: (*) *p* < 0.05, (**) *p* < 0.01, (***) *p* < 0.001 and (****) *p* < 0.0001.

To further investigate the pathogenesis of AD within the CNS, we aimed to clarify the role of tau protein within the hippocampal structure. To achieve this, we performed protein extraction from hippocampal tissue to enable subsequent Western blot analysis of phosphorylated tau isoforms. For that purpose, we used AT8 and AT100 antibodies. The AT8 antibody recognizes tau phosphorylated at serine 202 and threonine 205, which are sites within the microtubule-binding region of tau. The AT100 antibody, on the other hand, recognizes tau phosphorylated at threonine 212 and serine 214, which are also located within the microtubule-binding region of tau. Like AT8, the presence of phosphorylation at these sites has been linked to neurodegenerative diseases. Statistical analysis revealed that baseline levels of AT8 were similar between the Non-Tg and 5×FAD control groups, suggesting that 5×FAD mice may not be a reliable model of tauopathy in middle age. However, the 5×FAD-DPIN group led to a significant decrease in AT8 levels compared to the 5×FAD-CTR group (one-way ANOVA: *p* < 0.05; Figure 5A,E). Interestingly, treatment with DPIN in the Non-Tg group did not result in any significant changes in AT8 levels compared to its respective control group. In contrast, no significant changes were found in phosphorylated tau at Thr212-Ser214 measured using the AT100 antibody (one-way ANOVA: *p* = ns; Figure 5B,E) nor in total tau contents (one-way ANOVA: *p* = ns; Figure 5C,E) in any of the groups analyzed, regardless of DPIN treatment.

Another important hallmark of AD in the brain is the accumulation of Aβ. First, we measured the levels of the insulin-degrading enzyme (IDE), one of the main proteases that degrades Aβ in the brain, which is also responsible for preventing other b-forming peptide aggregates, including tau ones [65]. Our results revealed that DPIN significantly increased the expression of IDE in the 5×FAD group, indicating a promising mechanism for Aβ degradation (one-way ANOVA: *p* < 0.001; Figure 5D,E).

Additionally, our analysis showed a notable contrast between the Non-Tg and 5×FAD mice regarding Aβ plaque accumulation in the hippocampus. As shown in Figure 5F,H, the Non-Tg group of mice does not have traces of Aβ plaques in either the hippocampus. In contrast, 5×FAD-CTR mice displayed a substantial accumulation of plaques in the hippocampus (one-way ANOVA: *p* < 0.0001; Figure 5G,I). Importantly, DPIN treatment significantly reduced the number of Aβ1-40 (Figure 5F) and Aβ1-42 (Figure 5H) plaques in the hippocampus of the 5×FAD mice compared to the 5×FAD-CTR group (one-way ANOVA: *p* < 0.0001; Figure 5G,I).

### 3.6. Effects of D-Pinitol Treatment on Intestinal Barrier Function and Immune Response in the Small Intestine of 5×FAD Mice

**Figure 6:**
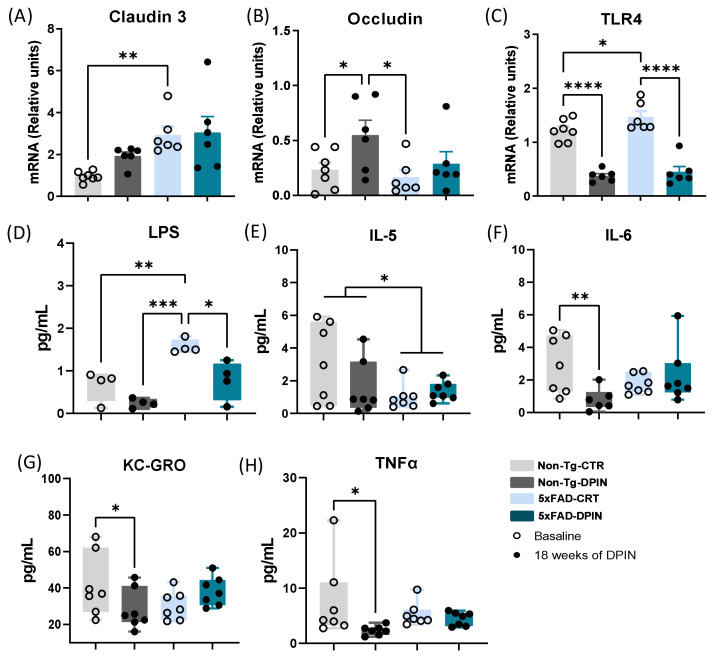
Effects of D-Pinitol treatment on proinflammatory cytokine levels in plasma and small intestine. mRNA expression (in relative units) in the small intestine of (**A**) Claudin 3, (**B**) occludin, and (**C**) Toll-like receptor 4 (TLR4). Graphs (**D**–**G**) correspond to plasma levels (pg/mL) of (**D**) LPS plasma level (pg/mL) and the pro-inflammatory cytokines (**E**) Interleukin 5 (IL-5), (**F**) Interleukin 6 (IL-6), (**G**) Keratinocyte chemoattractant (KC)/human growth-regulated oncogene (GRO), and (**H**) Tumor necrosis factor alpha (TNF-α). Histograms represent mean ± SEM (n = 7) in the groups Non-Tg and 5×FAD with (DPIN) and without (controls = CTR) D-Pinitol treatment. Two-way ANOVA and Tukey’s test for multiple comparisons were performed: (*) *p* < 0.05, (**) *p* < 0.01, (***) *p* < 0.001, and (****) *p* < 0.0001.

To analyze the effects of DPIN on constituents of the disrupted intestinal barrier of 5×FAD mice, we analyzed the expression of claudin-3 and occluding, as well as the expression of TLR4 eventually activated by leaked LPS from the gut. Statistical analysis showed a significant increase in Claudin-3 levels in the small intestine in the 5×FAD-CTR group (one-way ANOVA: *p* < 0.01; Figure 6A). Western blot results confirm these PCR findings (Supplementary Information (SI) Figure S4). However, following DPIN treatment in 5×FAD mice, those differences were not statistically significant, meaning that DPIN was restoring gene expression levels in 5×FAD mice.

Our results indicated that the occludin gene expression was significantly increased in Non-Tg-DPIN mice compared to the Non-Tg-CTR group (one-way ANOVA: *p* < 0.05; Figure 6B). Western blot results confirm these PCR findings (Supplementary Information (SI) Figure S4). 5×FAD-CTR mice exhibited significantly reduced levels of occludin gene expression compared to Non-Tg-DPIN mice. Conversely, following DPIN treatment in 5×FAD mice, these results were reversed, showing no significant differences in occludin gene expression compared to Non-Tg-DPIN mice.

To further analyze the impact of these intestinal barrier protein alterations and the potential beneficial action of DPIN administration, we measured both TLR4 expression, as a marker of activation of natural immunity, and LPS presence, as a marker of a leaky gut. Data show that TLR4 was highly expressed in the small intestine in the control group of both genotypes, being significantly higher in the 5×FAD compared to the Non-Tg (one-way ANOVA: *p* < 0.0001; Figure 6C). However, when DPIN treatment was administered, in both 5×FAD and non-transgenic mice, the expression of TLR4 was significantly decreased. Western blot results confirmed these PCR findings (Supplementary Information (SI) Figure S4). This activation of TLR4 might indicate the existence of a leaky gut, a fact confirmed by LPS analysis. LPS was also evaluated in plasma after 18 weeks of DPIN treatment. Statistical analysis revealed that the 5×FAD-CTR group exhibited significantly higher plasma levels of LPS compared to the Non-Tg-CTR group (one-way ANOVA: *p* < 0.001; Figure 6D). After DPIN treatment, LPS levels in plasma were reduced in the Non-Tg-DPIN group and 5×FAD-DPIN groups compared to their controls, respectively.

Plasma levels of various proinflammatory cytokines such as IL-5, IL-6, KC-GRO, and TNFα were also measured after DPIN treatment. Differences in the plasma levels of proinflammatory cytokines between 5×FAD and Non-Tg mice were observed. Specifically, 5×FAD mice had lower levels of IL-5 compared to Non-Tg mice, regardless of whether they received DPIN treatment or not (two-way ANOVA; ‘genotype’ factor: *p* < 0.05; Figure 6E). Non-transgenic mice that received DPIN treatment for 18 weeks had lower levels of IL-6, KC-GRO, and TNFα compared to Non-Tg-CTR mice (one-way ANOVA: *p* < 0.05; Figure 6F–H).

### 3.7. Differences in Fecal Microbiota by Genotype and D-Pinitol Treatment

**Figure 7:**
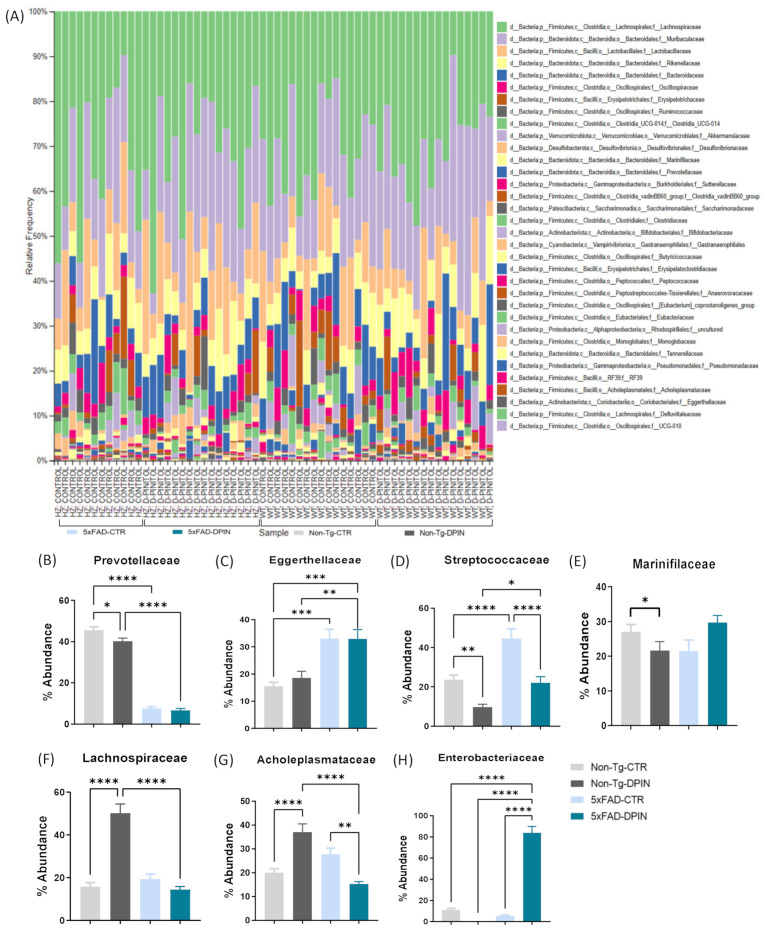
Differences in fecal microbiota by genotype and D-Pinitol treatment in Alzheimer’s transgenic and Non-Tg mice. (**A**) Taxonomic compositions obtained from the analysis of DNA sequences from fecal microbiota samples using QIIME2 (https://qiime2.org/, accessed on 1 January 2024) were compared at the family level in terms of relative frequency (%). The sequences were grouped into operational taxonomic units (OTUs) using a 97% similarity threshold. Significant differences for ‘Genotype × Treatment’ variables have been detected mostly in seven families: (**B**) Prevotellaceae, (**C**) Eggerthellaceae, (**D**) Streptococcaceae, (**E**) Marinifilaceae, (**F**) Lachnospiraceae, (**G**) Acholeplasmataceae, and (**H**) Enterococcaceae. Histograms represent relative abundance (%) in the groups Non-Tg and 5×FAD with (DPIN) and without (controls = CTR) D-Pinitol treatment. Statistical inference was performed using the Kruskal–Wallis test and Mann–Whitney U for each OTU, allowing for comparisons and identification of significant differences between groups: (*) *p* < 0.05, (**) *p* < 0.01, (***) *p* < 0.001, and (****) *p* < 0.0001.

The taxonomic compositions obtained from the analyses of DNA sequences extracted from fecal microbiota samples were analyzed using QIIME2. This comparison was performed at the family level in terms of relative abundance. Figure 7A displays abundance bars for each fecal sample. Significantly noticeable differences for the ‘Genotype × Treatment’ variables were predominantly detected within seven families.

Differences in *Prevotellaceae* abundance were found between Non-Tg and 5×FAD genotypes at the age of 8 months, irrespective of the treatment (one-way ANOVA: *p* < 0.0001; Figure 7B). More specifically, the Non-Tg genotype had a higher abundance of *Prevotellaceae*, which belongs to the Bacteroidetes phylum, compared to the 5×FAD genotype. 5×FAD mice showed a higher abundance of *Eggerthellaceae* (from the Actinobacteria phylum; one-way ANOVA: *p* < 0.0001; Figure 7C) and *Streptococcaceae* (from the Firmicutes phylum; one-way ANOVA: *p* < 0.0001; Figure 7D) compared to non-transgenic mice. Surprisingly, DPIN treatment for 8 weeks reduced the abundance of *Streptococcaceae* in the Non-Tg-DPIN and 5×FAD-DPIN groups compared to their controls. Therefore, the differences found in Figure 7B,C are due to the transgenic background, while in Figure 7D, they are mainly due to the treatment.

In addition, the DPIN treatment also had an impact on other bacterial populations. In the case of Non-Tg mice, the abundance of *Marinifilaceae*, belonging to the Bacteroidetes phylum, was only reduced in Non-Tg-DPIN (one-way ANOVA: *p* < 0.05; Figure 7E). In contrast, the abundance of *Lachnospiraceae* and *Acholeplasmataceae* was increased by DPIN treatment in the Non-Tg genotype (one-way ANOVA: *p* < 0.0001; Figure 7F,G) compared to its control. However, in the case of the 5×FAD genotype, DPIN treatment did not affect the abundance of *Lachnospiraceae* but reduced the abundance of *Acholeplasmataceae* compared to 5×FAD-CTR. This finding suggests a possible interaction between the *Acholeplasmataceae* population and the treatment, and it might be modulated by the genetic background of the host. Furthermore, DPIN treatment in 5×FAD mice had a higher impact on the abundance of *Enterobacteriaceae*, increasing the growth or colonization in the gut compared to the rest of the groups (one-way ANOVA: *p* < 0.0001; Figure 7H).

Analysis at the genus level (Supplementary Information (SI) Figure S5) showed differences in microbiota between Non-Tg and 5×FAD mice. Genotypic differences (without treatment) were observed in the *Eubacterium* genus (*Eubacteriaceae* family) and *Escherichia-Shigella* (two bacterial genera belonging to the *Enterobacteriaceae* family), with a higher population in 5×FAD.

Considering the DPIN effect, distinct impacts on Non-Tg and 5×FAD groups were detected. In the *Odoribacter* genus (*Odoribacteraceae* family), DPIN specifically affected the Non-Tg-DPIN group, leading to a decrease. Conversely, the population of *Clostridia vadinBB60* (*Clostridiaceae* family) was increased in the Non-Tg-DPIN group. In the case of *Escherichia-Shigella*, DPIN specifically influences the 5×FAD group. 5×FAD control animals exhibited a significantly higher abundance of *Escherichia-Shigella* compared to Non-Tg-CTR, although remarkably decreased with DPIN to levels comparable to Non-Tg-CTR was observed. *Streptococcus* (*Streptococcaceae* family) serves as a clear example of treatment impact, showing an increase in both Non-Tg and 5×FAD treated with DPIN compared to their controls. The rise was significantly greater in Non-Tg-DPIN than in 5×FAD-DPIN. This finding highlights the potential of DPIN to induce distinct responses in different genotypes, contributing to a more balanced microbiota composition.

## 4. Discussion

Our study explored the therapeutic potential of DPIN in addressing multidimensional alterations associated with AD using the transgenic 5×FAD mouse model. In this model, we observed Aβ deposits in the telencephalic regions, accompanied by changes in the insulin signaling cascade, indicative of brain insulin resistance. These central alterations were accompanied by cognitive impairment, including learning deficits, reduced cognitive flexibility, and deficient sensory assessment of stimuli. Additionally, 5×FAD mice exhibited metabolic hormone imbalances (insulin and glucagon), modifications in intestinal barrier protein modifications linked to bacterial LPS permeation, and altered gut microbiota, contributing to proinflammatory conditions. Oral DPIN treatment mitigated most of these alterations, reducing tau phosphorylation via CDK5R1 modulation and enhancing pro-cognitive hormones such as leptin or ghrelin. These findings suggest DPN administration may modify disease progression (Supplementary Information (SI) Figure S6). However, while promising, these effects were observed in a model that does not fully replicate human AD and animals with specific mutations, highlighting the need to consider DPIN as a disease modifier within these parameters.

The first point of discussion is the impact of DPIN on emotional behavior and cognitive function. Anhedonia, the inability to experience pleasure, is frequently reported in AD patients and has been linked to elevated cortical Aβ accumulation [66,67,68]. In our study (Figure 1), 5×FAD mice treated with DPIN for 18 weeks exhibited increased sucrose intake compared to their baseline values, suggesting improved reward behavior. DPIN also ameliorated atypical behavior observed in the EPM. Previous reports reported that 5×FAD mice spend more time in the open arms, avoiding the closed arms, which deviates from typical rodent behavior [54,64]. This abnormal behavior may be due to sensorimotor deficits or impaired vibrissal sensation, hindering their ability to assess the risks in the EPM [64]. Increased exploratory behavior in the EPM has been linked with Aβ accumulation [69,70,71], resembling the disinhibitory tendencies seen in AD patients [72].

The hippocampus, critical for memory and spatial navigation, is one of the earliest regions affected in AD. A well-established correlation between Aβ plaques and cognitive impairment [73] supports the memory improvements observed following DPIN treatment. In the MWM (Figure 2), both Non-Tg and 5×FAD mice treated with DPIN exhibited improved performance in reaching the visible platform during visual training compared to control groups. Notably, Non-Tg mice treated with DPIN demonstrated significantly faster escape latencies on the fourth day of acquisition training, indicating enhanced learning ability. After the acquisition phase, all groups showed a clear preference for the goal quadrant during the memory retention test 1. Some experimental protocols include a reversal phase, where the platform’s position is changed, challenging the animals to adapt to new spatial contexts [74]. In this task, hippocampal function is particularly crucial for relearning and distinguishing between different environments [74]. Previous research indicated that 5×FAD homozygous mice exhibit impaired cognitive flexibility, with slower relearning in reversal tasks due to an increase in Aβ deposits throughout the hippocampus [54]. In our study, 5×FAD mice treated with DPIN reached the hidden platform more quickly than controls during the reversal task, suggesting that DPIN improved their cognitive flexibility, possibly by reducing Aβ deposits in the hippocampus.

On the other hand, oral DPIN treatment showed notable effects on AD neuropathology, particularly through its impact on biochemical pathways. DPIN reduced Aβ deposits and tau levels in the hippocampus of 5×FAD mice, likely through increased IDE levels (Figure 5). IDE is a critical metalloprotease responsible for breaking down both insulin and amyloid β-protein. The competition between insulin and amyloid β-protein for IDE binding sites plays a key role in this process [75]. When IDE function is impaired, insulin degradation is affected, leading to insulin resistance and contributing to amyloid β-protein accumulation in the brain. This connection establishes a mechanistic link between insulin metabolism and AD pathology, emphasizing the central role of IDE in both insulin regulation and AD development [76,77].

One of the key biochemical pathways influenced by DPIN is the PI3K/Akt cascade, a critical cell signaling pathway that governs cell growth, survival, metabolism, and proliferation. This pathway is activated by insulin [47]. Our findings (Figure 4) indicated impaired PI3K/Akt pathway function in the hippocampus of 5×FAD mice, which was improved following DPIN treatment, as evidenced by increased phosphorylation of PI3K and Akt. This is significant because the PI3K/Akt cascade modulates Aβ production by influencing amyloidogenic pathways and tau hyperphosphorylation. Specifically, the PI3K/Akt pathway downregulates two key proteins involved in Aβ production—APP and Beta-site Amyloid Precursor Protein Cleaving Enzyme 1 (BACE-1). When the PI3K/Akt signalling pathway is active, APP and BACE-1 production is reduced, leading to decreasing Aβ production and a protective effect against AD progression [78,79]. This aligns with the observed impact of DPIN in our study.

Although the 5×FAD mice did not exhibit tau hyperphosphorylation within the examined age range, DPIN treatment specifically reduced tau phosphorylation at serine 202 and threonine 205, as detected by the AT8 antibody (Figure 5). These phosphorylation sites are closely linked to neurodegenerative diseases, particularly AD. In a previous study, DPIN’s effect on tau phosphorylation was explored in Wistar rats, particularly within insulin-mediated pathways [41]. Surprisingly, oral DPIN treatment significantly reduced tau phosphorylation, but not via the expected GSK3β kinase [80]. Instead, the reduction was mediated by decreased CDK5 activity [81], attributed to a marked decline in CDK5 activator 1 (CDK5R1) levels. This reduction, in turn, affected the CDK5 activator isoforms p35 and p25, responsible for activating CDK5. A similar reduction in tau phosphorylation was observed in a tauopathy model using 3xTg mice [41], confirming that CDK5 plays a key factor in AD pathology by phosphorylating tau at sites recognized by AT8. Our study extends these findings to the 5×FAD model, confirming that DPIN reduces tau phosphorylation in a manner consistent with results from Wistar rats and 3xTg mice. This demonstrates the efficacy of oral DPIN treatment in reducing AD-associated tauopathy. Beyond decreasing phosphorylated tau, DPIN also lowered elevated p35 levels in the hippocampus, which is a known driver of CDK5 activity (Figure 4). After 18 weeks of DPIN treatment, significant reductions in Aβ deposits, phosphorylated tau, and the P35/CDK5 ratio were observed in 5×FAD mice, suggesting DPIN’s potential in addressing critical neuropathological features of AD. In line with these findings, a study by Wilkaniec and colleagues identified CDK5 activation as an initiating factor in Aβ-induced neuroinflammation within the mouse hippocampus. Their research demonstrated that intracerebroventricular administration of Aβ1–42 led to a pronounced increase of the astrocytic (GFAP) and microglial (Iba-1) markers, as well as heightened cytokine synthesis, suggesting that Aβ-induced CDK5 activation significantly impacts reactive gliosis [82]. These results reinforce the role of CDK5 in the inflammatory processes associated with AD, providing further context for DPIN’s efficacy in modulating this pathway.

In addition to these findings, we explored the roles of metabolism-regulating hormones and inflammation in contributing to cognitive decline and AD progression (Figure 3). Notably, 5×FAD mice showed altered insulin and glucagon levels, with DPIN treatment normalizing insulin levels. Surprisingly, DPIN also increased leptin and ghrelin—two hormones involved in energy homeostasis with pro-cognitive effects [83,84]. This suggests that DPIN may activate metabolic mechanisms that support cognition, though further research is needed to confirm this. While an increase in ghrelin following DPIN treatment has been observed in both rats and humans, its effect on leptin remains undocumented [42,44]. DPIN also downregulated Plasminogen Activator Inhibitor-1 (PAI-1), a significant protein implicated in AD progression. PAI-1 plays a critical role in fibrinolysis and is associated with cellular senescence in the brain, contributing to AD progression [85,86,87]. Its absence is linked to reduced amyloid burden and cognitive impairment in AD mouse models. By downregulating PAI-1 in both Non-Tg and 5×FAD mice, DPIN likely contributed to the improved behavioral and neuropathological outcomes observed in treated animals.

Inflammation is a hallmark of AD, driven not only by proinflammatory Aβ species but also by bacterial products and immune signals from the gut that can cross the BBB. The ‘leaky gut’ hypothesis suggests that bacterial products and immune signals from a disrupted intestinal barrier may accelerate AD progression [88]. In this sense, our study revealed that 5×FAD displayed increased intestinal permeability, evidenced by elevated levels of LPS, which triggered an inflammatory response via TLR4 activation (Figure 6). This likely stemmed from dysregulation of intestinal barrier proteins such as claudin-3 and occludin [89]. Claudin-3, a tight junction protein, regulates intestinal permeability, while occludin plays a critical role in maintaining the integrity of the intestinal barrier. Excessive claudin-3 expression can lead to increased intestinal permeability, potentially allowing harmful substances to pass through, whereas higher occludin levels generally promote intestinal health [90,91]. A decrease in TLR4 expression following DPIN treatment, along with reduced LPS levels, suggests that DPIN may modulate the immune response. These findings indicate that oral DPIN treatment helps restore intestinal barrier integrity, reducing inflammation as evidenced by lower cytokine levels.

DPIN also demonstrated a significant effect on gut microbiota composition (Figure 7). The gut microbiota profile varied depending on the presence of the human transgene (5×FAD) or DPIN treatment. The family *Prevotellaceae*, known for breaking down complex carbohydrates and dietary fibers [92], was more abundant in Non-Tg mice than in 5×FAD mice, suggesting healthier and more diverse gut microbiota in Non-Tg animals. Conversely, 5×FAD mice had a higher abundance of *Eggerthellaceae* and *Streptococcaceae*, both associated with inflammation, indicating a more inflammatory gut environment in these mice. Surprisingly, oral DPIN treatment notably reduced *Streptococcaceae* abundance in both Non-Tg and 5×FAD groups compared to controls, suggesting a potential anti-inflammatory effect. Elevated *Enterobacteriaceae* levels in 5×FAD mice may reflect an inflammatory response linked to the underlying AD pathology. However, the changes in the *Enterobacteriaceae* population following DPIN treatment warrant further investigation to better understand its role in gut health and AD progression.

Regarding the genus, we observed significant differences between Non-Tg and 5×FAD mice, regardless of treatment. 5×FAD mice exhibited higher levels of the *Eubacterium* and *Escherichia-Shigella* genera, both of which are associated with inflammation and disease progression [93]. Conversely, they had lower levels of the *Clostridium* genus, known for its role in promoting gut health [94]. Interestingly, after 18 weeks of DPIN treatment, 5×FAD mice showed a reduction in the *Odoribacter* genus and an increase in the *Clostridia* genus, indicating shifts toward a more balanced microbiota. Moreover, DPIN reduced *Escherichia-Shigella* genus levels in both Non-Tg and 5×FAD groups, suggesting that the treatment’s effects were more pronounced in 5×FAD mice.

These findings highlight that the gut microbiota of the 5×FAD mice was more responsive to DPIN treatment compared to Non-Tg mice, likely due to the greater inflammatory burden associated with AD pathology. In summary, the investigation uncovered significant compositional differences in gut microbial families between the two genotypes at 8 months of age. DPIN treatment contributed to a more balanced microbiota, particularly in 5×FAD mice, suggesting its potential to modulate gut health and alleviate inflammation linked to AD. These changes in gut bacteria correlate with reductions in LPS and TLR4 levels, as well as inflammatory markers in plasma. In AD, the activation of the TLR4 pathway contributes to intestinal inflammation and compromises the intestinal barrier. Additionally, AD-related genes elevate serum LPS levels and promote bacterial translocation, which may exacerbate intestinal diseases. DPIN administration appeared to mitigate these detrimental effects, reducing intestinal inflammation and restoring barrier integrity in 5×FAD mice.

A key limitation of this study is the use of a 5×FAD model, which, while effective for studying accelerated amyloidosis, does not encompass all characteristics of Alzheimer’s disease, particularly in sporadic cases. This model focuses on mutations in two key proteins—APP and PSEN-1—central to Aβ plaque formation, which limits its generalizability to all forms of AD. Despite this limitation, the pharmacological profile of DPIN supports its potential use in mitigating various factors that contribute to cognitive impairment, particularly in the early stages of the disease. Furthermore, these results position DPIN as a potential disease modifier and highlight its utility as a tool for the development of new AD treatments. DPIN could be combined with other therapeutic strategies, such as immunotherapies targeting Aβ, to enhance treatment outcomes.

## 5. Conclusions

This study highlights the potential of D-Pinitol (DPIN) as a disease-modifying agent for Alzheimer’s disease (AD). In the 5×FAD mouse model, DPIN treatment mitigated key pathological features of AD, including Aβ deposition, tau hyperphosphorylation, and impaired insulin signaling in the hippocampus. These effects were accompanied by improved cognitive function, particularly in learning, memory, and cognitive flexibility. Mechanistically, DPIN modulated the PI3K/Akt signaling cascade, reduced CDK5 activity, and normalized levels of metabolic hormones such as insulin, leptin, and ghrelin. Additionally, DPIN enhanced intestinal barrier integrity and induced significant shifts in gut microbiota composition, reducing pro-inflammatory bacterial populations and systemic inflammation. Although the 5×FAD model provides valuable insights into early-sonset amyloidosis, it does not fully replicate the heterogeneity of human AD, particularly in sporadic cases. Despite this limitation, this study positions DPIN as a promising candidate for addressing the multifactorial nature of AD and provides a foundation for its development as a therapeutic tool.

## Data Availability

A detailed protocol outlining the research question, key design features, and analysis plan was prepared before the commencement of the study. This protocol was not registered in any public repository. This published article and its Supplementary Information files include all data generated or analyzed during this study. Raw data are available upon reasonable request from the corresponding author.

## Supplementary Materials

The following supporting information can be downloaded at https://www.mdpi.com/article/10.3390/nu16234186/s1. Table S1: Primary antibodies used for protein expression by Western blotting. Figure S1: Behavioral learning procedure. Figure S2: Whole membranes from Gel 1 (unedited blots). Figure S3: Whole membranes from Gel 2 (unedited blots). Figure S4: Whole membrane from Gel 3 (unedited blots). Figure S5: Differences in fecal microbiota by genotype and D-Pinitol treatment in Alzheimer’s transgenic and Non-Tg mice. Figure S6: Mechanistic diagram to provide a holistic understanding of the proposed therapeutic effects of D-Pinitol in Alzheimer’s Disease.
