# Supplementary material for: Therapeutic Efficacy of the Inositol D-Pinitol as a Multi-Faceted Disease Modifier in the 5×FAD Humanized Mouse Model of Alzheimer’s Amyloidosis
Source: Nutrients. 2024 Dec 4;16(23):4186. doi: 10.3390/nu16234186 (PMC11644622; doi:10.3390/nu16234186)
Supplement: Supplementary file 1 [file nutrients-16-04186-s001.zip › nutrients-3328438-supplementary.pdf]

Supplementary Table S1:

Primary antibodies used for protein expression by Western blotting.

ANTIGEN	MANUFACTURING DETAILS	DILUTION	Phosphorilation action
CDK5 (1H3)	Cell Signaling Technology (#12134)	1:1000	
p35/25 (C64B10)	Cell Signaling Technology (#2680)	1:1000	
GAMMA-ADAPTIN	bd biosciences (#610385)	1:2000	
PHOSPHO-GSK3 BETA (SER9) [D85E12]	Cell Signaling Technology (#5558)	1:1000	Inhibitory
GSK3 BETA [D5C5Z]	Cell Signaling Technology (#12456)	1:1500	
PHOSPHO-AKT (SER473)	Cell Signaling Technology (#9271)	1:1000	Activator
AKT	Cell Signaling Technology (#9272)	1:1000	
PHOSPHO-PI3K-P85 (TYR607)	Abcam (#182651)	1:1000	Activator
PI3K-P85 [19H8]	Cell Signaling Technology (#4257)	1:1000	
Claudin 3	Thermo Fisher Scientific # 34-1700	1:1000	
Occludin	Thermo Fisher Scientific # OC-3F10	1:1000	
TLR4	Abcam (#217274)	1:1000	
PHOSPHO-Tau (Ser202, Thr205) [AT8]	Thermo Fisher Scientific (#MN1020)	1:1000	
PHOSPHO-Tau (Thr212, Ser214) [AT100]	Thermo Fisher Scientific (#MN1060)	1:1000	
Tau [Tau46]	Abcam (#ab203179)	1:1000	
IDE	Abcam (#ab32216)	1:1000	

Abbreviations: ; GSK3β, glycogen synthase kinase 3 beta; MTOR, mammalian target of rapamycin; TLR4: toll-like receptor 4; IDE: insulin-degrading enzyme (IDE) enzyme

Supplementary Figure S1:

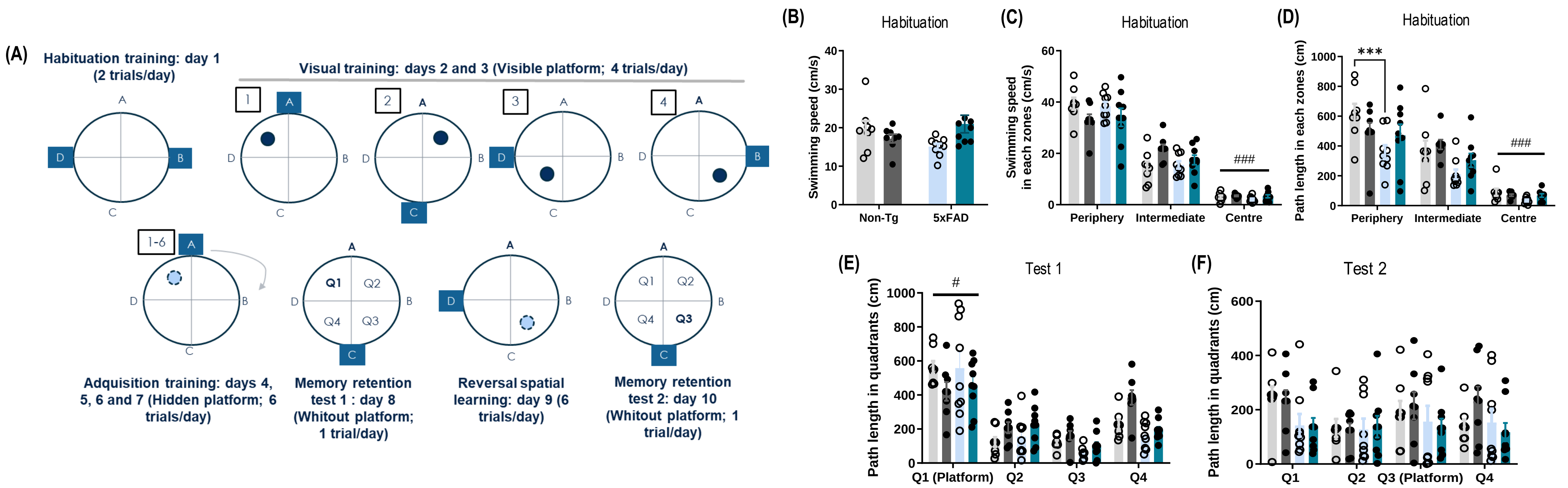

(A) Behavioural learning procedure: Habituation training: Mice received 2 trials for one day and were allowed to explore the pool freely for 60 s. The pool was divided into three zones: periphery (20 cm from walls), intermediate (from 20 cm to 50 cm from walls) and centre (50 cm from walls). Path length and swimming speed were analysed. **Visual training:** Mice received 4 trials for two days (30-min intertribal intervals). The trial ended when the mouse climbed into the visible platform or after the maximum trial duration of 60 s. If a mouse did not find the platform during a trial, it was placed on the platform for 10 s. The platform's location and the animals' starting point were changed in each trial. The escape latency, path length, cumulative distance to reach the platform and swimming speed were analysed. **Acquisition training:** Animals received 6 trials for four days (30-min intertribal intervals). The hidden platform was located in quadrant 1 (Q1) during all training. The starting point of mice was changed in each trial. The maximum duration of the trial was 60 s unless the mouse reached the hidden platform. We analysed the escape latency, path length, cumulative distance to reach the platform and swimming speed. **Memory retention test 1:** Twenty-four hours after the last trial of acquisition training, mice received one trial without a platform. The duration of the trials was 60 s. The time spent in the target quadrant (Q1) and the other quadrants as well as the path length in each one was examined. **Reversal spatial learning:** Mice received 6 trials for one day (30-min intertribal intervals). The hidden platform was positioned in quadrant 3 (Q3) (opposite to its placement on acquisition training days). The escape latency and cumulative distance to reach the platform were evaluated. **Memory retention test 2:** Twenty-four hours after the last trial of reversal spatial learning, mice swam for the 60s without the platform. The time spent on each quadrant and path length was analysed. **(B)** Swimming speed in centimetres/seconds (cm/s) during the habituation training. Each subject received two trials per day. All groups showed a significantly longer path length (cm) **(C)** and swimming speed (cm/s) **(D)** in the peripheral zone than in the central zone (### p<0.0001 central zone vs peripheral zone, *** p<0.0001) during the habituation training. **(E)** On memory retention test 1 all animals demonstrated higher path length (cm) on the target quadrant (Q1) (# p<0.05 Q1 vs the other quadrants). **(F)** On memory retention test 2, no differences between groups were noticed Regarding the path length (cm).

Supplementary Figure S2:

GEL 1

Ponceau S stain

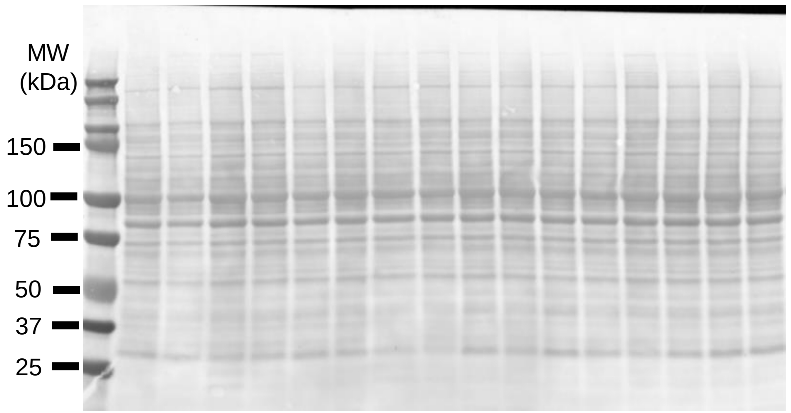

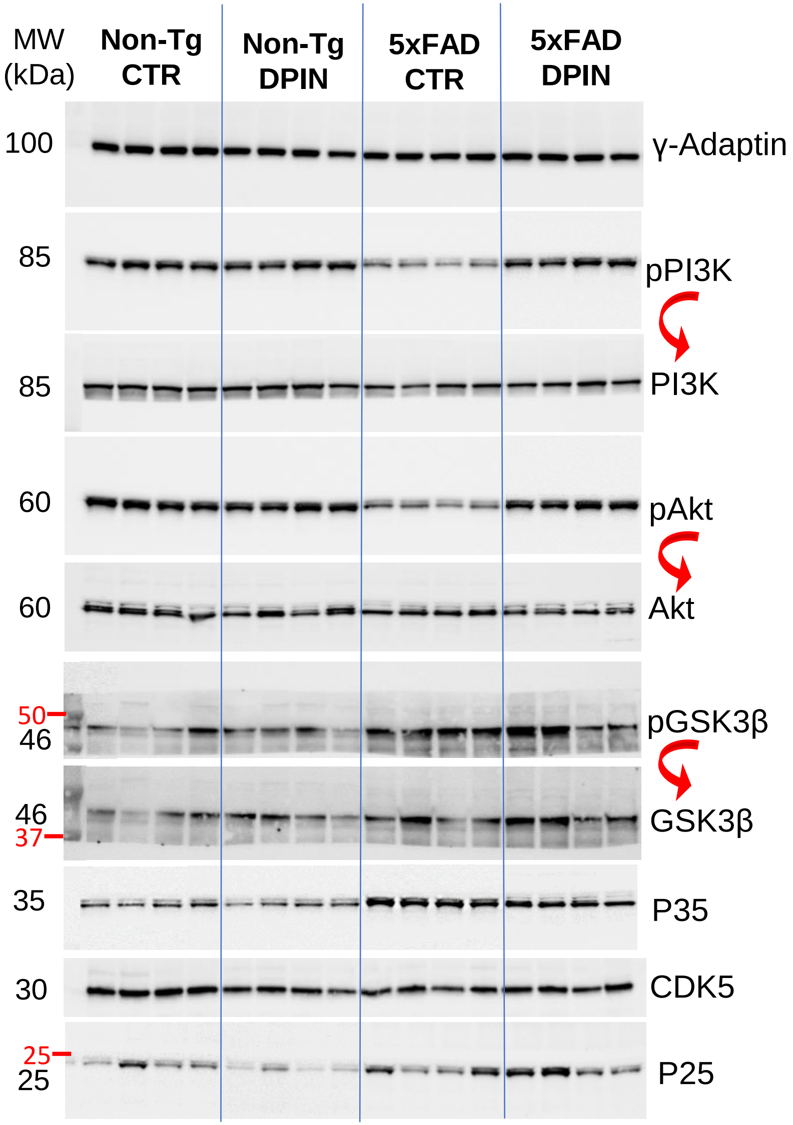

Whole membranes from Gel 1 (unedited blots). One membrane was used for immunoblottings. Red Ponceau Staining and individual membranes are shown below. All bands were quantified for histograms charts and statistical analysis. Proteins were normalized with its respective γ-Adaptin. Molecular weights (MW) are indicated in kilodaltons (kDa) of the studied proteins; in red, the molecular weight signals of the gel. The red arrows indicate a stripping process between one and the other protein indicated.

Supplementary Figure S3:

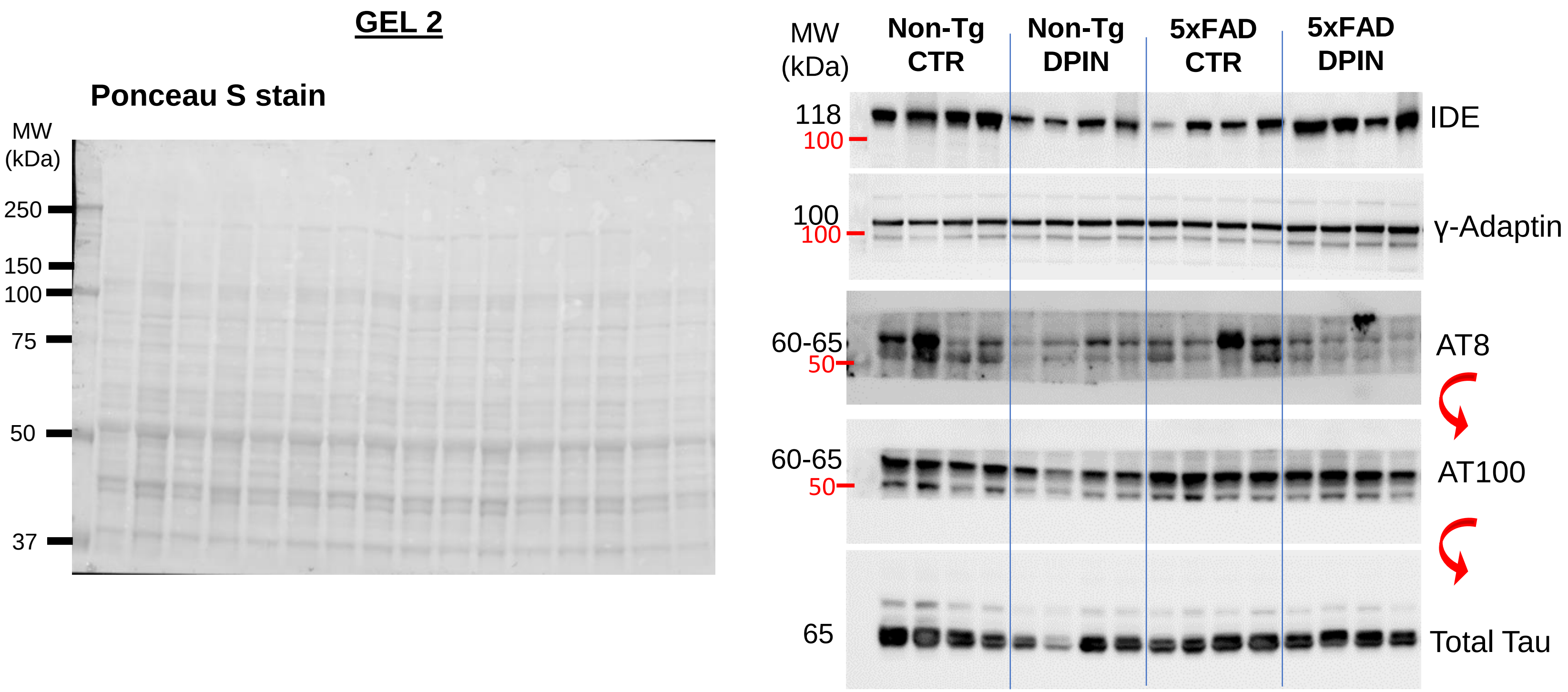

Whole membranes from Gel 2 (unedited blots). One membrane was used for immunoblottings of insulin-degrading enzyme (IDE), tau [AT8] phosphorylation on serine 202 and threonine 205 (p-Tau(S202/T205)), tau [AT100] phosphorylation on threonine 212 and serine 214 (p-tau(T212/S214)) and total tau. Red Ponceau Staining and individual membranes are shown below. All bands were quantified for histograms charts and statistical analysis. Proteins were normalized with its respective γ-Adaptin. Molecular weights (MW) are indicated in kilodaltons (kDa) of the studied proteins; in red, the molecular weight signals of the gel. The red arrows indicate a stripping process between one and the other protein indicated.

Supplementary Figure S4:

GEL 3

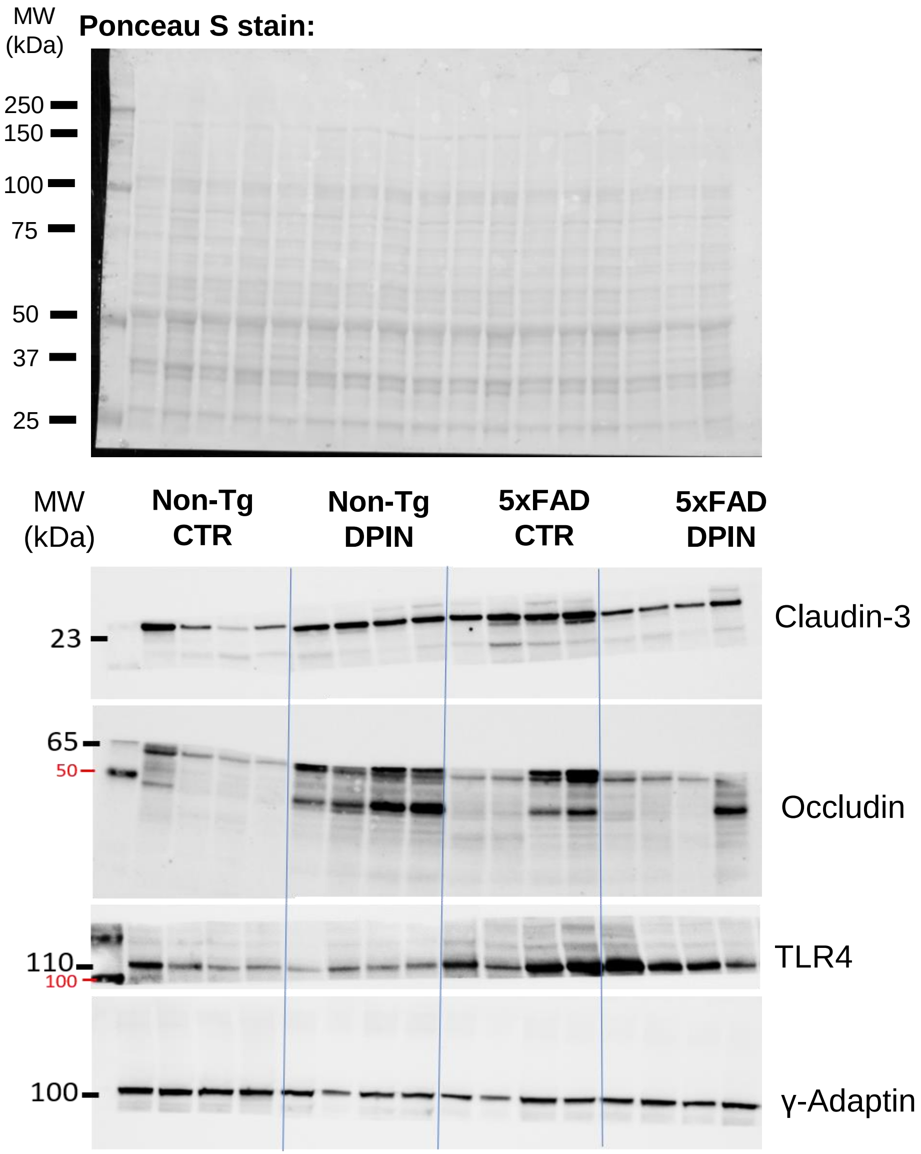

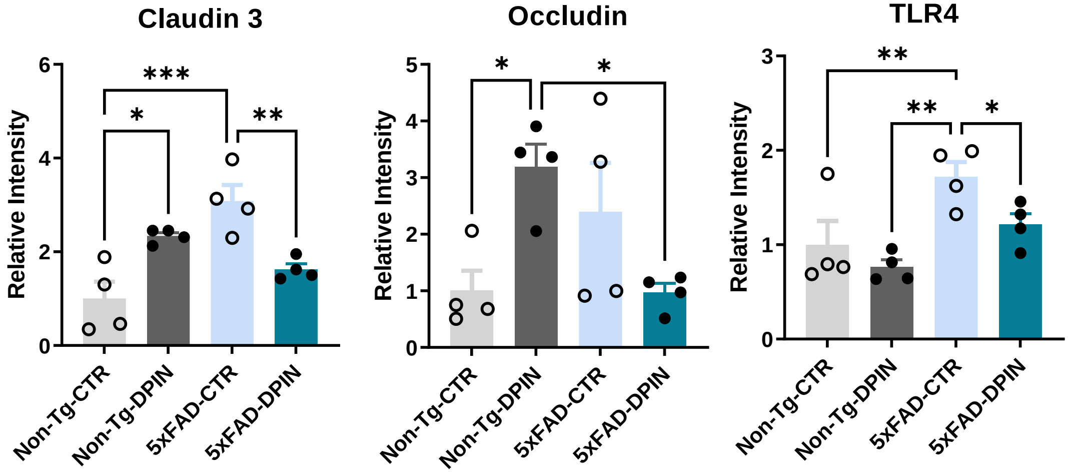

Whole membrane (unedited blots). One membrane was used for immunoblotting of small intestine tissue samples. Red Ponceau Staining and individual membranes are shown below. All bands were quantified for histogram charts and statistical analysis. Proteins were normalized with its respective γ-Adaptin. Molecular weights (MW) are indicated in kilodaltons (kD) of the studied proteins; in red, the molecular weight signals of the gel. Groups: Non-Tg and 5xFAD with (DPIN) and without (controls = CTR) D-Pinitol treatment. Two-way ANOVA and Tukey's test for multiple comparisons were performed: (*) $p<0.05$, (**) $p<0.01$ and (***) $p<0.001$. Abbreviations: Toll-like receptor 4 (TLR4).

Supplementary Figure S5:

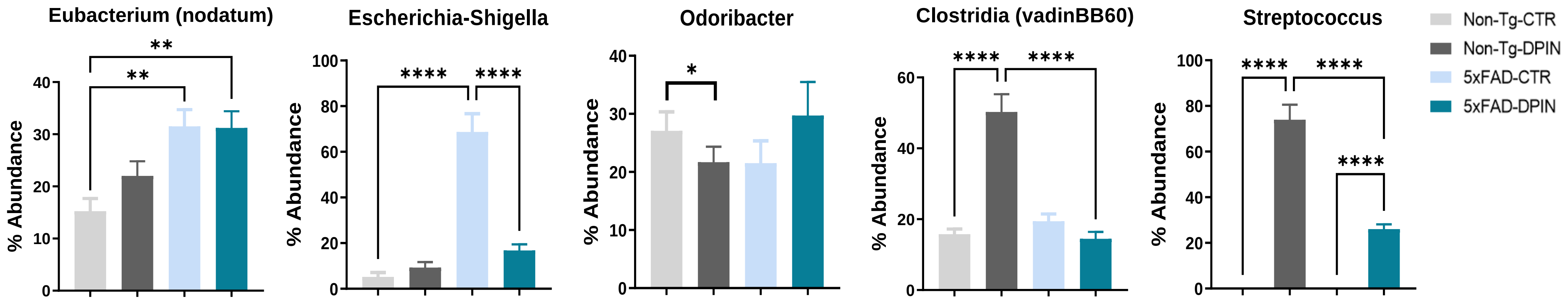

Differences in faecal microbiota by genotype and D-Pinitol treatment in Alzheimer's transgenic and Non-Tg mice. Taxonomic compositions obtained from the analyses of sequences of DNA from faecal microbiota samples using QIIME2 (https://qiime2.org/) were compared at the genus level in terms of relative frequency (%). Histograms represent relative abundance (%) in the groups Non-Tg and 5xFAD with (DPIN) and without (controls = CTR) D-Pinitol treatment. Statistical inference was performed using the Kruskal–Wallis test and Mann-Whitney U for each OTUs, allowing for comparisons and identification of significant differences between groups: (*) P < 0.05, (**) P < 0.01, (***) and P < 0.001.

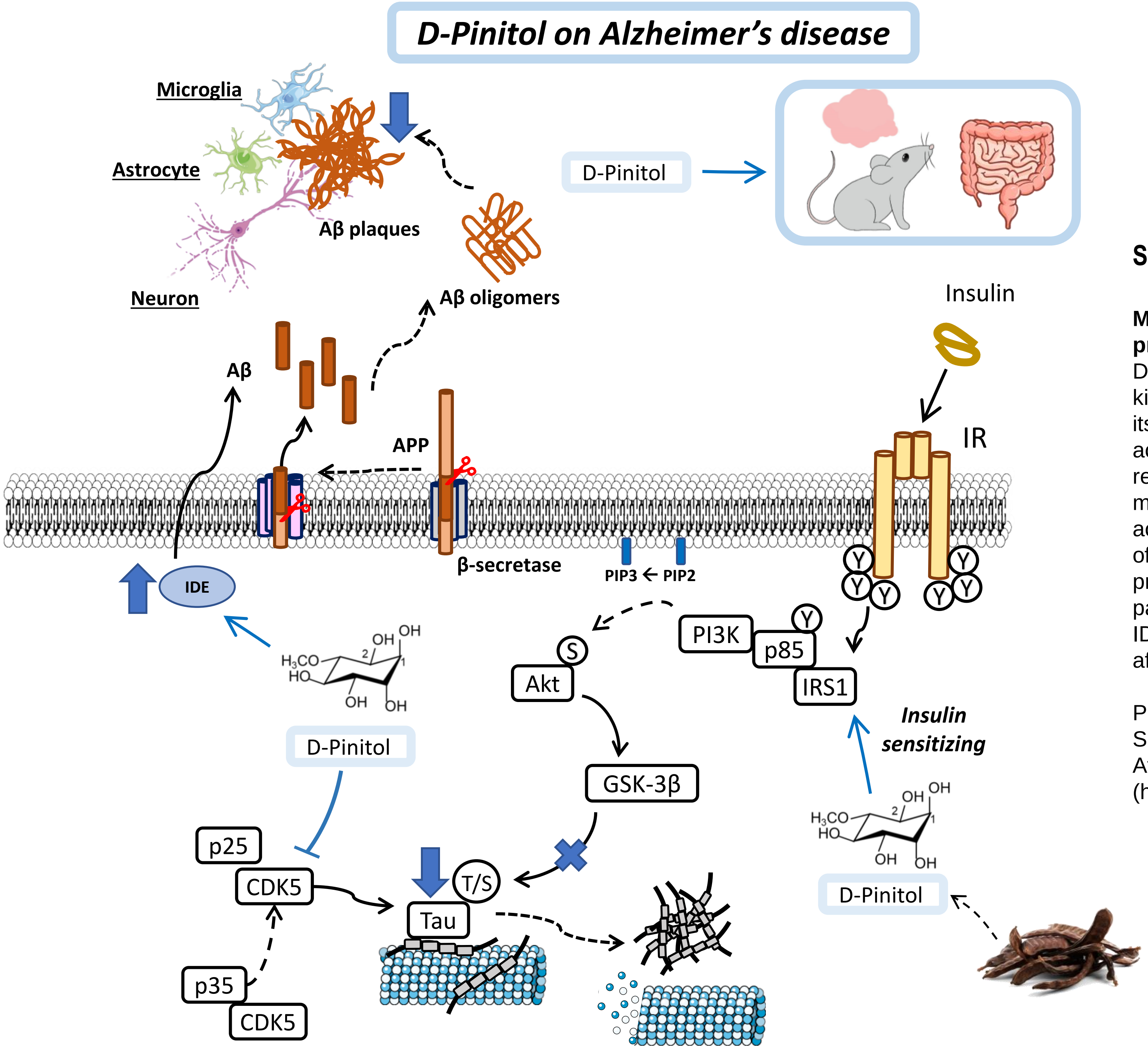

Supplementary Figure S6:

Mechanistic diagram to provide a holistic understanding of the proposed therapeutic effects of D-Pinitol in Alzheimer's Disease. D-Pinitol inhibits specifically pathogenic activation of tau-activating kinase, cyclin-dependent kinase 5 (CDK5), preventing the cleavage of its bound protein cyclin-dependent kinase 5 activator 1 (p35) into active cleaved cyclin-dependent kinase 5 activator 1 (p25), thus reducing tubulin-associated unit (Tau) hyperphosphorylation and microtubule destabilization. On the other hand, D-Pinitol is also able to activate insulin receptor substrate 1 (IRS1) and decrease the activation of glycogen synthase protein kinase-3β (GSK-3β), which also promotes Tau hyperphosphorylation. D-Pinitol is also able to reduce pathogenic amyloid β (Aβ) fragment formation, likely through increased IDE levels (insulin degradation enzyme). Futhermore, D-Pinitol is affecting mice behaviour, microbiota and immflamation.

Parts of the figure were drawn using pictures from Servier Medical Art. Servier Medical Art by Servier is licensed under a Creative Commons Attribution 3.0 Unported License (https://creativecommons.org/licenses/by/3.0/).
